# Acute Upper Gastrointestinal Bleeding: A Hands-On Simulation Case for Internal Medicine Residents Improves Knowledge and Confidence

**DOI:** 10.15766/mep_2374-8265.11541

**Published:** 2025-08-01

**Authors:** Marni H. Wilkoff, Emily S. Seltzer, Nicholas R. Piniella, Harrindra Seepersaud, Priscilla Loanzon, Susannah Kurtz, James Salonia, Daniela Jodorkovsky

**Affiliations:** 1 Resident, Department of Medicine, Icahn School of Medicine at Mount Sinai, Mount Sinai Morningside and West; 2 Resident, Department of Orthopedic Surgery, RWJ Barnabas, Jersey City Medical Center; 3 Simulation Specialist and Research Assistant, Division of Pulmonary, Critical Care and Sleep Medicine, Icahn School of Medicine at Mount Sinai, Mount Sinai West; 4 CAMS Director of Simulation Education, Division of Pulmonary, Critical Care and Sleep Medicine, Icahn School of Medicine at Mount Sinai, Mount Sinai West; 5 Associate Professor, Division of Pulmonary, Critical Care and Sleep Medicine, Icahn School of Medicine at Mount Sinai, Mount Sinai West; 6 Director, Medical and Surgical Intensive Care Unit, Division of Pulmonary, Critical Care and Sleep Medicine, Icahn School of Medicine at Mount Sinai, Mount Sinai West; 7 Associate Professor, Division of Gastroenterology, Icahn School of Medicine at Mount Sinai, Mount Sinai Morningside and West; Associate Professor, Department of Medical Education, Icahn School of Medicine at Mount Sinai

**Keywords:** Gastroenterology, Simulation, Internal Medicine, Case-Based Learning

## Abstract

**Introduction:**

Upper gastrointestinal (GI) bleeding leads to approximately 350,000 hospital admissions annually. Simulation-based training enhances medical education by improving quality care, patient safety, and clinical competency. To increase internal medicine (IM) residents’ exposure to critical GI concepts, we developed a GI bleed simulation curriculum.

**Methods:**

A total of 129 IM residents participated in a hands-on simulation using a high-fidelity manikin. Pre- and postsimulation surveys assessed demographics, confidence, and knowledge. The case involved a 45-year-old male with alcohol use disorder, hematemesis, and hemodynamic instability. Key learning outcomes included assessing vitals, performing a physical exam, initiating resuscitation, ordering appropriate medication, consulting GI, and creating a differential. Critical equipment included a code cart and moulage blood. A postsimulation debrief addressed the management of esophageal varices (EV), peptic ulcer disease (PUD), central venous access, massive transfusion protocol, and hemorrhagic shock.

**Results:**

Confidence improved for PGY 1 and PGY 2 residents in all categories (*p* < .05). PGY 3 residents increased their confidence managing EV (*p* = .03), PUD (*p* = .002), and outpatient EV (*p* = .003). PGY 1 and PGY 2 knowledge increased with treatment of nonvariceal GI bleeds (*p* < .001, *p* = .001). All residents increased in their knowledge of timing of endoscopy for EV bleeds (*p* < .001). Among all residents combined, there was an increase in knowledge of discharge medications for EV and PUD (*p* = .01).

**Discussion:**

A hands-on simulation curriculum positively impacted IM residents’ confidence and knowledge in managing GI bleeds, highlighting its educational value.

## Educational Objectives

By the end of this session, learners should be able to:

1. Create a differential diagnosis for upper gastrointestinal (GI) bleeds.
2. List the indications for massive transfusion protocol in an unstable upper GI bleed.
3. Formulate the stages of hemorrhagic shock.
4. Learn appropriate medical management of upper GI bleeds and timing of upper endoscopy.
5. Describe proper inpatient management of upper GI bleeds.

## Introduction

In recent years, simulation-based training (SBT) has become an exceedingly popular medical education tool, with notable enhancement of quality care and patient safety.^1,2^ SBT can be performed on a variety of devices, including manikins, virtual-reality simulators, plastic models, standardized patients, or screen-based simulators.^1^ Low-fidelity simulators are low cost and less lifelike, whereas high-fidelity simulators replicate patients’ physiologic responses and recreate clinical scenarios.^3,4^ In a controlled environment, simulation helps learners recognize a disease process, and initiate proper workup and treatment.^5–7^

As of 2011, 64 participating teaching hospitals utilized simulation for residents in various specialties including internal medicine, emergency medicine, general surgery, pediatrics, anesthesiology, and obstetrics-gynecology.^5^ Of these teaching hospitals, 61% used simulation for subspecialty training and four of the top five subspecialties fall under internal medicine and include critical care, pulmonology, cardiology, and GI, with 31% of the hospitals having screen-based virtual reality simulation for GI endoscopic skills.^5^ Studies involving low- and high-fidelity simulators in GI fellowship have been performed and have been shown to improve fellow knowledge and endoscopic skills.^8–10^ However, data are lacking on utility of GI-based simulation in internal medicine residents.

Upper gastrointestinal (GI) bleeding is a common cause of hospitalization in the United States, accounting for approximately 350,000 admissions annually, with general internal medicine physicians being responsible for routine care of patients hospitalized for GI bleeds.^11,12^ Optimal management prior to endoscopic intervention includes resuscitation, medical management with proton pump inhibitors or vasoactive agents when appropriate, understanding the role of endoscopic intervention, and appropriate discharge planning.^13–18^

SBT is a requirement for the GME training of internal medicine residents.^19^ A needs assessment was distributed to all 151 internal medicine residents at our institution during the 2022–2023 academic year, with a 21% response rate. Results showed that 75% felt they would benefit from additional hands-on experience with GI topics. This prompted the development of a pilot educational innovation using a hands-on high-fidelity GI endoscopy simulator supplementing a low-fidelity educational lecture. Positive feedback from the pilot educational initiative prompted expansion of the curriculum to the entire residency class focused on the topic of GI bleed using high-fidelity simulation. The aim of our educational innovation was to determine whether use of a simulation curriculum increased confidence and medical knowledge in the management of GI bleed.

## Methods

### Development

We developed a hands-on case-based simulation, with simulation faculty (Harrindra Seepersaud, Priscilla Loanzon, Susannah Kurtz, and James Salonia) and gastroenterology faculty (Daniela Jodorkovsky) serving as simulation and content experts, respectively. During the session, we covered evidenced-based recommendations, which included management of bleeding and nonbleeding esophageal varices (EV), variceal screening guidelines, peptic ulcer disease (PUD), proton pump inhibitor (PPI) use, appropriate timing for upper endoscopy, central venous access, hemorrhagic shock, and outpatient management of EV and PUD.^13–18,20,21^ No prerequisite knowledge was required for the learners, though knowledge on management of GI bleeds was helpful.

We designed a case involving a 45-year-old male with a past medical history of alcohol use disorder and chronic back pain who presented to the emergency department with one week of abdominal pain and swelling with progression of the case detailed by changing vital signs and physical exam. During the simulation, a rapid response was called 12 hours after admission for hematemesis associated with hemodynamic instability. At each time point, we outlined expected learner actions and modifiers, which included need for venous access, resuscitation with packed red blood cell transfusion, intubation, and endoscopic intervention. We created a case outline detailing a narrative description of the case, learning objectives, critical actions, history of present illness, vital signs on admission and time of deterioration, overall patient appearance, past medical and surgical history, patient medications, allergies, family history and physical exam (Appendix A).

### Equipment/Environment

We conducted the simulation at the Center for Advanced Medical Simulation (CAMS) center at the Mount Sinai West Hospital using a SimMan 3G Manikin. The simulation faculty positioned the manikin on a stretcher and was covered with a clean blanket. Faculty provided materials, which included a code cart, monitor/defibrillator, nasal cannula, nonrebreather mask, bag-valve-mask (BVM), cardiac monitor, peripheral IVs, blood tubes, facemask with moulage blood (red marker), chuck with moulage melena (black and red marker), emesis basin with moulage hematemesis (coffee grounds, water, gelatin, red food coloring), crystalloid, central line and introducer kits, and intubation equipment. Learners accessed patient's history of present illness, medical history, labs and images using a computer in the simulation room (Appendix B).

### Personnel

A total of 138 internal medicine residents at a single residency program were included. Learners consisted of interns (PGY1) and second- (PGY2) and third-year (PGY3) residents in a single residency program. The residents designated roles for each other including a team leader, obtaining a history, physical exam, calling consultants, and airway. Simulation faculty and staff, pulmonary and critical care board-certified physicians, and case authors (Marni Wilkoff and Emily Seltzer) acted as confederates, including a registered nurse (RN), GI fellow, and critical care physician. We conducted a brief run through of the simulation prior to each session. The simulation faculty had advanced knowledge with operating the SimMan 3G Manikin and changed vital signs and communicated through the mannequin as needed.

### Implementation

We conducted the simulation sessions on Tuesdays and Thursdays afternoons over an 8-week period. Each session lasted 1 hour and included four to six residents. We began each session with a prebrief, which oriented the residents to important details regarding the upcoming simulation including situation details, equipment available to them, and roles of confederates. Prior to entering the simulation lab, an anonymous presimulation REDCap knowledge assessment was administered, which included demographic, confidence, and knowledge assessment questions (Appendix C). Upon completion of the REDCap assessment, the learners were moved to the simulation lab, where they were greeted by a nurse who activated the rapid response team for tachycardia and worsening abdominal pain. The learners were expected to complete a full physical exam, assess vital signs, order appropriate labs, diagnostic tests, transfusion products, medications, and call consultants.

History was obtained from the PowerPoint containing the HPI and patient's medical history as well as the simulation operator via the manikin speaker (Appendix B). The patient could be heard retching through the manikin speaker but would not voluntarily inform the learners that he was having hematemesis or melena. While the learners were obtaining a history and physical exam, the nurse would reveal a basin with moulage hematemesis. If not already done, the learners were expected to remove the blanket covering the manikin and find moulage melena on the stretcher.

The patient became hemodynamically unstable throughout the case, which prompted learners to order blood tests, blood products, PPI, octreotide, and consultations from the ICU and GI. Notable labs revealed a blood urea nitrogen (BUN) of 29 mg/dL, creatinine (Cr) 0.9 mg/dL, hemoglobin 6.4 g/dL, hematocrit 19.2%, platelet 82 K/uL, pH 7.28, lactate 3.1 mmol/L, and international normalized ratio (INR) 2.8. The learners were able to request an EKG, right upper quadrant ultrasound, computerized tomography of the abdomen and pelvis and a chest X-ray. Upon transfer to the ICU, GI arrived and performed a bedside endoscopy, which was simulated via two videos showing PUD, EV, and endoscopic treatment. After GI performed the endoscopy, the patient was transferred to the general medical floor. The case concluded when the team ordered appropriate discharge medications for the patient including a nonselective beta blocker and PPI twice daily. The simulation lasted about 20–25 minutes. If the simulation went over the allotted time, the session was stopped, with follow-up discussion occurring in the debrief. A faculty guide outlining how to conduct the simulation can be found in Appendix D.

### Debriefing

We conducted a 30–40 minute debrief at the end of the simulation, which included a PowerPoint lecture discussing both inpatient and outpatient management of EV and PUD, central venous access, massive transfusion protocol, and hemorrhagic shock, all topics encountered during the simulation (Appendix E). The case authors (Marni Wilkoff and Emily Seltzer) led the debrief and were supported by board-certified physicians and simulation faculty. We asked each group to reflect on their experience with the simulation and to provide suggestions on how they could improve their performance. In instances where the simulation exceeded the allotted time, we invited residents to reflect on potential contributing factors—such as knowledge gaps, time management challenges, or ineffective task delegation, among others—that may have hindered completion. Faculty facilitators participated in the debriefing sessions, reviewed the checklist, and discussed which key tasks were successfully executed or overlooked, as well as strategies for improvement moving forward.

We opted for a lecture-based PowerPoint format to present visual aids and display information clearly on the screen, making it easier for learners to engage in discussions and formulate questions. Questions posed to learners included whether they understood the stages of hemorrhagic shock, Forrest Classification, and treatment options, including correct dosing for inpatient and outpatient management of PUD and EV. Prior to conclusion of the debrief, we asked learners to state one point they learned from session and to complete the postsimulation assessment.

### Assessment

Simulation faculty assessed learners based on predefined critical actions (Appendices A and F), which were identified as lifesaving or medically necessary interventions. The case authors (Marni Wilkoff and Emily Seltzer) created a 16-question presimulation REDCap knowledge assessment, which was administered immediately before the simulation and included demographic, confidence level (rated using a 4-point Likert scale [1 = *not confident*, 2 = *somewhat confident*, 3 = *moderately confident*, 4 = *extremely confident*]), and knowledge assessment questions, which were based on information obtained through thorough literature review and were not previously tested for validity.^13–18,20,21^ Simulation faculty completed the observational checklist (Appendix F). Upon conclusion of the simulation and debrief, we administered an anonymous postsimulation knowledge assessment using the same questions as the presimulation assessment (Appendix D). Additional questions were available at the end of the postsimulation assessment, which asked for nonmandatory feedback regarding the session and included free-text questions, allowing participants to write their thoughts about the session (Appendix C).

We used chi-square test and ANOVA test for continuous variables to compare pre- and postsimulation confidence, knowledge improvement, and clinical question accuracy when appropriate. A *p* value less than .05 was considered statistically significant. This educational innovation was determined exempt by the the Icahn School of Medicine at Mount Sinai Institutional Review Board (STUDY-23-00492, date of exemption: 7/20/2023; STUDY-23-00492-MOD002, date of exemption: 1/21/2024).

## Results

There were 138 categorical residents in the program during the time of the simulation, which was used 16 times over an 8-week period during the 2023–2024 academic year. There was a 93% response rate presimulation (129/138) and 86% response rate postsimulation (118/138). Of those who completed the preassessment, 36% were PGY1, 33% were PGY2, and 30% were PGY3. Prior to the simulation, 10% of learners were interested in pursuing a GI fellowship, 40% previously rotated on the GI service, 91% had prior experience treating GI bleeds, 63% had prior experience treating esophageal varices, and 85% had prior experience treating PUD. For the postassessment, 38% were PGY1, 35% were PGY2, and 27% were PGY3. All faculty members work in medical education. Subsequent use of this simulation will be used by gastroenterology faculty, critical care faculty, and internal medicine residents.

PGY1 and PGY2 residents had a statistically significant increase in their knowledge postsimulation with regards to treatment of nonvariceal GI bleeds (PGY 1: *p* < .001, PGY 2: *p* = .001; Table 1). While there was an improvement in PGY 1 and PGY 2 knowledge in treating acute variceal hemorrhage, the results were not significant (Table 1). Notably, PGY 3 knowledge decreased slightly from 84.6% to 81.3% with regards to acute variceal hemorrhage and from 100% to 96.9% for nonvariceal GI bleed treatment (Table 1). All residents had a statistically significant increase in their knowledge of the timing of endoscopy for acute esophageal variceal bleeds postsimulation (*p* < .001; Table 1). While individual resident levels did not show a statistically significant increase in their knowledge of discharge medications for EV and PUD, there was an overall significant increase with all residents combined (*p* = .01; Table 1).

**Table 1.**
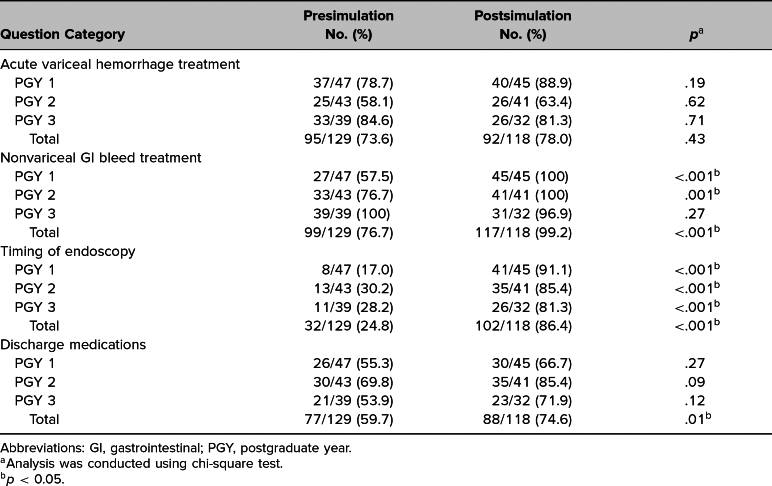
Analysis of Resident Correct Questions of GI Bleed Case Survey

There was a significant increase in the level of confidence pre- and postsimulation for PGY 1 and PGY 2 residents in all categories (*p* < .05; Table 2). PGY 3 residents had a significant increase in confidence in managing acute variceal bleeds (*p* = .03), peptic ulcer bleeding (*p* = .002), and outpatient EV (*p* = .003; Table 2).

**Table 2.**
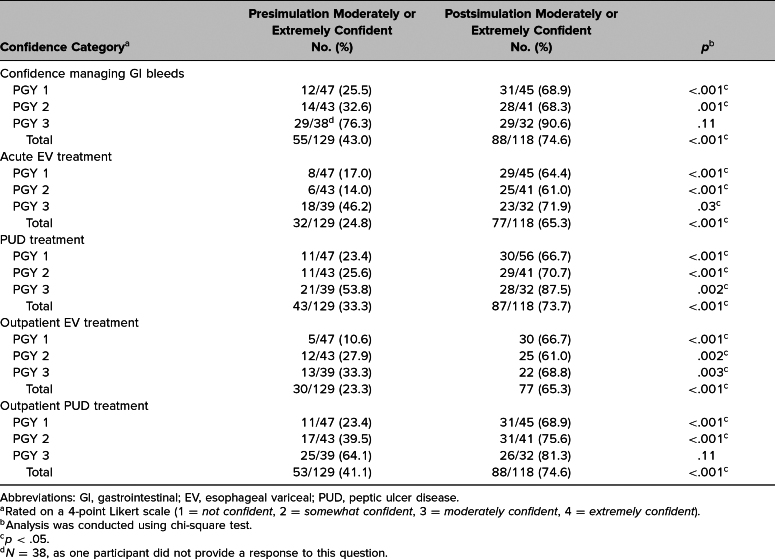
Analysis of Resident Moderate/Extreme Confidence Pre- and Postsimulation

Both PGY level (*p* < .001) and interest in GI (*p* = .001) had a significant positive effect on knowledge improvement and confidence level.

Select comments highlighting benefits of the simulation case are included below:

• “Great session. Straight to the point and rapid review.”
• “Was great learning about alternative IV access.”
• “Excellent and helpful case.”

## Discussion

Simulation has become a prevalent tool used in medical education and this educational innovation demonstrates the feasibility of creating and implementing a GI bleeding curriculum amongst residents. The use of a hands-on high-fidelity manikin simulation had a positive impact on internal medicine resident confidence and knowledge managing GI bleeds and underscores the importance of individual characteristics in influencing learner outcomes. In addition to measurable gains in knowledge and confidence, learners reported high levels of satisfaction with the simulation experience. Although learning preferences may vary, simulation provides learners with real-time hands-on clinical scenarios that change with their medical decision-making, compared to lecture alone. These scenarios provide real-time feedback, helping the learner improve in a safe environment.

The positive association between PGY level and confidence suggests that more experienced residents tend to exhibit higher confidence levels, likely attributed to their accumulated knowledge and familiarity with the material, but PGY 3 knowledge dropped slightly with regards to variceal and nonvariceal bleed treatment, possibly due to low engagement or interest in the simulation, as it may not relate to their future career goals. Similarly, the positive relationship between interest in GI as a specialty and confidence implies that learners with a greater interest in the subject matter tend to feel more at ease, potentially due to proactively reading or seeking experience. These insights highlight the potential benefits of tailoring educational innovations to align with learners' experience levels and interests, facilitating enhanced engagement and effectiveness in educational outcomes.

Strengths of this educational innovation include the large sample size, with residents of all PGY levels participating. Selection bias was eliminated, as this simulation was integrated into the IM resident curriculum and was required of all trainees. Limitations of the educational innovation included lack of a comparator arm that would assess differences between simulation format and lecture-only format, although in our pilot educational initiative, we found that residents who chose the simulation plus lecture had both a statistically significant increase in their overall confidence and confidence with PUD treatment compared to those who participated in lecture alone. Another limitation to our educational innovation was that while we found medical knowledge statistically improved in the postsimulation survey, there was a lack of longitudinal testing to confirm retention of material and knowledge. The knowledge assessment questions were not previously tested and were missing validity evidence, and confidence questions were asked using a subjective Likert scale, so it is unclear how much the score must improve to indicate a meaningful outcome. Finally, this simulation required significant faculty involvement and availability as well as complex simulation equipment, which could be a constraining factor for institutions with limited faculty or equipment.

This simulation will be incorporated into the residency curriculum moving forward with the plans to integrate longitudinal knowledge assessment. A key lesson was the importance of tailoring simulations to reflect real-world clinical challenges, to maximize lasting educational impact. Moving forward, we will use this experience to design simulations that address specific gaps in medical knowledge and foster teamwork, critical thinking, and decision-making skills in a safe, controlled environment.

This was a proof-of-concept initiative that showed a GI bleed simulation could be incorporated into the IM residency program curriculum. Along with fulfilling the ACGME simulation requirement, simulation appears to improve confidence and medical knowledge in GI topics.

## Appendices


Simulation Case.docxPatient HPI, Labs, and Imaging.pptxPre- and Postsimulation Surveys.docxFaculty Guide.docxDebriefing.pptxCritical Action Checklist.docx
*All appendices are peer reviewed as integral parts of the Original Publication.*
