# Supplementary material for: Acute Upper Gastrointestinal Bleeding: A Hands-On Simulation Case for Internal Medicine Residents Improves Knowledge and Confidence
Source: MedEdPORTAL. 2025 Aug 1;21:11541. doi: 10.15766/mep_2374-8265.11541 (PMC12313986; doi:10.15766/mep_2374-8265.11541)
Supplement: Supplementary file 1 — Simulation Case.docxPatient HPI, Labs, and Imaging.pptxPre- and Postsimulation Surveys.docxFaculty Guide.docxDebriefing.pptxCritical Action Checklist.docx [file mep_2374-8265.11541-s001.zip › C. Pre- and Postsimulation Surveys.docx]

**Pre-Training Clinical Event Debriefing Survey Questionnaire**

Name (Optional)

Email:

Today’s Date

**Introduction:** Clinical event debriefing is a learning tool through team conversations that happen immediately among those involved after clinical patient care encounters. Clinical event debriefing intends to improve future care and team performance.

**Instructions:** Please answer the questions that best reflect your thoughts and or experience related to clinical event debriefing. You may be asked to select one response from available choices or answer open-ended questions.

**Demographics:** Please provide the following information:

**Objective of this question/item:** To help correlate career and the level of GME training with the subject’s participation, experience, knowledge, skills, and abilities related to GI bleeding simulation.

Assumption 1: The higher the level of GME training, the more knowledge, skills, and abilities will be demonstrated by the participants.

1. Level of Post-Graduate Medical Education
   1. Intern
   2. PGY 2
   3. PGY 3
2. Are you interested in pursuing a GI fellowship?
   1. Yes
   2. No
   3. Unsure

**Objective of this question/item:** to determine baseline participation and experience of the subjects.

Assumption 2: The role and participation of the subjects can be used as metrics to determine the quality of the experience. The role and rate of participation of the subjects intersect with the knowledge, skills, and abilities (KSA) of the subjects before the simulation-based training. The higher the role in the Team hierarchy, the higher the level of KSA.

1. Have you ever rotated on the GI service?
   1. Yes, once
   2. Yes, multiple times
   3. No
2. Have you had experience treating GI bleeds?
   1. Yes, rarely
   2. Yes, often
   3. No
3. Have you had experience treating bleeding esophageal varices?
   1. Yes
   2. No
4. Have you had experience treating esophageal varices?
   1. Yes, outpatient only
   2. Yes, inpatient only
   3. Yes, both inpatient and outpatient
   4. No
5. Have you had experience treating peptic ulcer disease?
   1. Yes, outpatient only
   2. Yes, inpatient only
   3. Yes, both inpatient and outpatient
   4. No

**Objective of this question/item:** To validate the answer to question and assumption # 2.

Assumption 2: The role and rate of participation of the subjects can be used as metrics to determine the quality of the experience. The role and experience of the subjects intersect with the knowledge, skills, and abilities (KSA) of the subjects. The higher the role in the Team hierarchy, the higher the level of KSA.

**The overall objective of the following questions/items** is to determine the subject’s concepts and perception of their skills, and abilities to diagnose and manage various causes of GI bleeds.

1. How comfortable are you managing GI bleeds?
   1. Not comfortable at all
   2. Slightly comfortable
   3. Moderately comfortable
   4. Extremely comfortable
2. How comfortable are you managing acute variceal bleeds?
   1. Not comfortable at all
   2. Slightly comfortable
   3. Moderately comfortable
   4. Extremely comfortable
3. How comfortable are you managing gastric/duodenal ulcer bleeds?
   1. Not comfortable at all
   2. Slightly comfortable
   3. Moderately comfortable
   4. Extremely comfortable
4. How comfortable are you with outpatient management of esophageal varices?
   1. Not comfortable at all
   2. Slightly comfortable
   3. Moderately comfortable
   4. Extremely comfortable
5. How comfortable are you with outpatient management of peptic ulcer disease?
   1. Not comfortable at all
   2. Slightly comfortable
   3. Moderately comfortable
   4. Extremely comfortable
6. A 45-yo male with a past medical history of cirrhosis complicated by small esophageal varices on endoscopy 2 years ago and chronic back pain presents to the emergency department with progressively worsening abdominal pain and swelling. Labs show a Hb of 9.3, AST 93, ALT 45, total bilirubin 1.8 and direct bilirubin 1.0. While you are admitting him to the medicine service, he develops acute hematemesis. Given his known history of cirrhosis and esophageal varices, which medication should be started?
   1. Propranolol
   2. Carvedilol
   3. **Octreotide**
   4. Pepcid
   5. Protonix
   6. Levophed
   7. None of the above
7. In order to treat other causes of an upper GI bleed, which of the following medications should be started in addition to the medication chosen above?
   1. Pepcid 20mg oral every 12 hours
   2. Protonix 40mg oral every 12 hours
   3. Protonix 40mg IV daily
   4. **Protonix 80mg IV bolus followed by 40mg IV every 12 hours**
8. The patient becomes hemodynamically unstable with tachycardia to 130 bpm and hypotension to 80/50. Repeat labs show a Hb of 6.4 down from 9.3 in the emergency room. He is given 1L IVF and 1u PRBC. GI is consulted for endoscopy and will perform the procedure when the patient is adequately resuscitated. When should the endoscopy be performed?
   1. Within 1 hour of acute hemorrhage
   2. Within 4 hours of acute hemorrhage
   3. Within 8 hours of acute hemorrhage
   4. **Within 12 hours of acute hemorrhage**
   5. Within 24 hours of acute hemorrhage
9. GI performs endoscopic ligation of the esophageal varices and also find a bleeding duodenal ulcer that is treated endoscopically. The patient is transferred to the general medical floor and remains stable throughout the duration of his hospitalization. What medication(s) should the patient be discharged on to prevent further bleeding episodes?
   1. Cardioselective beta blocker
   2. Nonselective beta blocker
   3. Pepcid twice a day
   4. PPI twice a day
   5. A and D
   6. A and C
   7. B and C
   8. **B and D**

**Post-Training Clinical Event Debriefing Survey Questionnaire**

Name (Optional)

Email:

Today’s Date

**Introduction:** Clinical event debriefing is a learning tool through team conversations that happen immediately among those involved after clinical patient care encounters. Clinical event debriefing intends to improve future care and team performance.

**Instructions:** Please answer the questions that best reflect your thoughts and or experience related to clinical event debriefing. You may be asked to select one response from available choices or answer open-ended questions.

**Demographics:** Please provide the following information:

**Objective of this question/item:** To help correlate career and the level of GME training with the subject’s participation, experience, knowledge, skills, and abilities related to GI bleeding simulation.

Assumption 1: The higher the level of GME training, the more knowledge, skills, and abilities will be demonstrated by the participants.

1. Level of Post-Graduate Medical Education
   1. Intern
   2. PGY 2
   3. PGY 3
2. Are you interested in pursuing a GI fellowship?
   1. Yes
   2. No
   3. Unsure

**Objective of this question/item:** to determine baseline participation and experience of the subjects.

Assumption 2: The role and participation of the subjects can be used as metrics to determine the quality of the experience. The role and rate of participation of the subjects intersect with the knowledge, skills, and abilities (KSA) of the subjects before the simulation-based training. The higher the role in the Team hierarchy, the higher the level of KSA.

1. Have you ever rotated on the GI service?
   1. Yes, once
   2. Yes, multiple times
   3. No
2. Have you had experience treating GI bleeds?
   1. Yes, rarely
   2. Yes, often
   3. No
3. Have you had experience treating bleeding esophageal varices?
   1. Yes
   2. No
4. Have you had experience treating esophageal varices?
   1. Yes, outpatient only
   2. Yes, inpatient only
   3. Yes, both inpatient and outpatient
   4. No
5. Have you had experience treating peptic ulcer disease?
   1. Yes, outpatient only
   2. Yes, inpatient only
   3. Yes, both inpatient and outpatient
   4. No

**Objective of this question/item:** To validate the answer to question and assumption # 2.

Assumption 2: The role and rate of participation of the subjects can be used as metrics to determine the quality of the experience. The role and experience of the subjects intersect with the knowledge, skills, and abilities (KSA) of the subjects. The higher the role in the Team hierarchy, the higher the level of KSA.

**The overall objective of the following questions/items** is to determine the subject’s concepts and perception of their skills, and abilities to diagnose and manage various causes of GI bleeds.

1. How comfortable are you managing GI bleeds?
   1. Not comfortable at all
   2. Slightly comfortable
   3. Moderately comfortable
   4. Extremely comfortable
2. How comfortable are you managing acute variceal bleeds?
   1. Not comfortable at all
   2. Slightly comfortable
   3. Moderately comfortable
   4. Extremely comfortable
3. How comfortable are you managing gastric/duodenal ulcer bleeds?
   1. Not comfortable at all
   2. Slightly comfortable
   3. Moderately comfortable
   4. Extremely comfortable
4. How comfortable are you with outpatient management of esophageal varices?
   1. Not comfortable at all
   2. Slightly comfortable
   3. Moderately comfortable
   4. Extremely comfortable
5. How comfortable are you with outpatient management of peptic ulcer disease?
   1. Not comfortable at all
   2. Slightly comfortable
   3. Moderately comfortable
   4. Extremely comfortable
6. A 45-yo male with a past medical history of cirrhosis complicated by small esophageal varices on endoscopy 2 years ago and chronic back pain presents to the emergency department with progressively worsening abdominal pain and swelling. Labs show a Hb of 9.3, AST 93, ALT 45, total bilirubin 1.8 and direct bilirubin 1.0. While you are admitting him to the medicine service, he develops acute hematemesis. Given his known history of cirrhosis and esophageal varices, which medication should be started?
   1. Propranolol
   2. Carvedilol
   3. **Octreotide**
   4. Pepcid
   5. Protonix
   6. Levophed
   7. None of the above
7. In order to treat other causes of an upper GI bleed, which of the following medications should be started in addition to the medication chosen above?
   1. Pepcid 20mg oral every 12 hours
   2. Protonix 40mg oral every 12 hours
   3. Protonix 40mg IV daily
   4. **Protonix 80mg IV bolus followed by 40mg IV every 12 hours**
8. The patient becomes hemodynamically unstable with tachycardia to 130 bpm and hypotension to 80/50. Repeat labs show a Hb of 6.4 down from 9.3 in the emergency room. He is given 1L IVF and 1u PRBC. GI is consulted for endoscopy and will perform the procedure when the patient is adequately resuscitated. When should the endoscopy be performed?
   1. Within 1 hour of acute hemorrhage
   2. Within 4 hours of acute hemorrhage
   3. Within 8 hours of acute hemorrhage
   4. **Within 12 hours of acute hemorrhage**
   5. Within 24 hours of acute hemorrhage
9. GI performs endoscopic ligation of the esophageal varices and also find a bleeding duodenal ulcer that is treated endoscopically. The patient is transferred to the general medical floor and remains stable throughout the duration of his hospitalization. What medication(s) should the patient be discharged on to prevent further bleeding episodes?
   1. Cardioselective beta blocker
   2. Nonselective beta blocker
   3. Pepcid twice a day
   4. PPI twice a day
   5. A and D
   6. A and C
   7. B and C
   8. **B and D**
10. Rate the effectiveness
    1. Not effective
    2. Somewhat effective
    3. Moderately effective
    4. Extremely effective
11. Satisfaction with learning experience
    1. Not satisfied
    2. Somewhat satisfied
    3. Satisfied
    4. Extremely satisfied
12. Did this course meet expectations
    1. Not met
    2. Somewhat met
    3. Met
    4. Exceeded expectations
13. Faculty educators answered questions
    1. Strongly agree
    2. Agree
    3. Somewhat agree
    4. Disagree
14. What would you change
    1. Time
    2. Debriefing methods
    3. Mannequin or supplies
    4. Room or physical space
    5. Course/content
    6. Other: ____________
15. Safe learning environment
    1. Strongly agree
    2. Agree
    3. Somewhat agree
    4. Disagree
16. Additional comments: ___________________
