# Supplementary material for: Acute Upper Gastrointestinal Bleeding: A Hands-On Simulation Case for Internal Medicine Residents Improves Knowledge and Confidence
Source: MedEdPORTAL. 2025 Aug 1;21:11541. doi: 10.15766/mep_2374-8265.11541 (PMC12313986; doi:10.15766/mep_2374-8265.11541)
Supplement: Supplementary file 1 — Simulation Case.docxPatient HPI, Labs, and Imaging.pptxPre- and Postsimulation Surveys.docxFaculty Guide.docxDebriefing.pptxCritical Action Checklist.docx [file mep_2374-8265.11541-s001.zip › D. Faculty Guide.docx]

**Title:** Acute Upper Gastrointestinal Bleeding: A Hands-On Simulation Case for Internal Medicine Residents Improves Knowledge and Confidence

**Location:** Simulation Lab
**Duration:** 1 hour
**Learners:** Internal Medicine Residents
**Faculty/Roles:**

- **Gastroenterologist faculty facilitator** (1): observe, act as GI consult fellow if available (can have simulation faculty act as consulting GI fellow if needed), assist with debrief session
- **Critical care physician faculty facilitator** (1): observe, act as ICU consult attending / anesthesiologist during intubation, assist with debrief session, act as lead debrief instructor
- **Simulation technician** (2): 1 person to control the manikin and vital signs, 1 person to control the monitor and show labs/imaging/videos when prompted
- **Simulation instructor** (1): lead the pre-simulation huddle, act as the covering nurse, assist with debrief
- **Learners** (4-6)**:** take on active roles in team-based simulation

**Pre-Simulation Preparation (30 minutes)**

| **Time** | **Activity** | **Lead** | **Notes** |
| --- | --- | --- | --- |
| -30 mins | Check equipment, setup, brief run through | Simulation technician and instructor | Manikin, monitors, props in place |
| -20 mins | Instructor team huddle | All facilitators | Review scenario script, faculty roles, and learner roles |
| -10 mins | Learner arrival and sign-in | Simulation instructor | Distribute pre-simulation knowledge assessment |
| -5 mins | Learner huddle | Simulation instructor | Set ground rules, confidentiality, expectations |
| -2 mins | Learner role assignment | Learners | Assign team leader, history obtainer, physical exam, airway |

**Simulation (25 minutes)**

| **Time** | **Segment** | **Lead** | **Technology** | **Notes** |
| --- | --- | --- | --- | --- |
| 0-2 min | **Arrival at rapid response** | Learners, simulation instructor | Activate patient manikin and vitals machine | Learners start to take a history and examine the patient. Patient retching. |
| 2-8 min | **Initial Assessment** | Learners, Simulation technician | Adjust vital signs; provide voice for manikin; monitor available for residents to view HPI and labs | Patient reveals the following information if asked: vomiting blood, drinks 6 beers a day for 20 years, takes ibuprofen daily. |
| 8-13 min | **Hemorrhagic shock** | Learners, simulation technician | Adjust vitals; labs result on monitor | Real-time manikin response to learner actions. RN reveals emesis basin if asked. Learners should order blood products, octreotide and ceftriaxone, and consult GI. If incorrect medications are ordered, GI consult fellow asks probing questions but does not reveal exact medication recommendations. |
| 13-16 min | **Intubation and ICU consult** | Learners, simulation technician, critical care physician | Adjust vitals; intubation equipment bedside | Learners should know to suction the mouth given prior hematemesis. Anesthesia called for intubation. ICU consulted for further management. |
| 16-22 min | **Transfer to ICU and endoscopy** | Learners, simulation technician, gastroenterologist | Adjust vitals; monitor available for showing endoscopy videos | Patient is admitted to the ICU (physical location of the manikin does not change, all activities remain in the simulation room). GI performs bedside endoscopy, and videos appear on the monitor for the learners to watch. |
| 22-25 min | **Stable on floors** | Learners, simulation technician | Adjust vitals; repeat labs result on monitor | Learners should order proper discharge medications including a nonselective beta block and PPI. |

**Debrief (35 minutes)**

| **Time** | **Activity** | **Lead** | **Notes** |
| --- | --- | --- | --- |
| 25-30 min | **Faculty-led Feedback** | Simulation instructor, critical care physician | Immediate learner reflection. Faculty and physicians ask about any performance gaps the learners noticed. Critical actions checklist reviewed. |
| 30-50 min | **Debrief PowerPoint** | Simulation instructor, critical care physician, gastroenterologist | Review PowerPoint. |
| 55 min-1 hr | **Wrap-Up and Takeaways** | Simulation instructor, critical care physician | Answer final questions. Learners state one learning objective they will take away. Post-simulation assessment distributed. |

**Potential Learner Questions:**

- Which beta blocker is preferred for secondary prophylaxis of variceal hemorrhage?
  - Carvedilol 6.25mg daily
- What is the re-bleeding risk for the different Forrest-classified ulcers?
  - Grade Ia: Actively spurting arterial bleed
    - Re-bleeding risk: 60-100%
  - Grade Ib: Actively oozing venous bleed
    - Re-bleeding risk: 50%
  - Grade IIa: Non-bleeding visible vessel
    - Re-bleeding risk: 40-50%
  - Grade IIb: Adherent clot
    - Re-bleeding risk: 20-30%
  - Grade IIc: Flat pigmented hematin spot on the ulcer base
    - Re-bleeding risk: 7-10%
  - Grade III: Clean-based ulcer with no signs of active bleeding
    - Re-bleeding risk: 3-5%
- Does PPI treatment duration change if the ulcer is in the stomach or duodenum?
  - Gastric ulcers: 4-6 weeks
  - Duodenal ulcers: 6-8 weeks
- Is there a mortality difference if you use intermittent high dose IV PPI v continuous?
  - No, either can be used
- What are the first stages of hemorrhagic shock?
  - Tachycardia. By the time the patient is hypotensive, they have lost a significant amount of blood.
