# Supplementary material for: Acute Upper Gastrointestinal Bleeding: A Hands-On Simulation Case for Internal Medicine Residents Improves Knowledge and Confidence
Source: MedEdPORTAL. 2025 Aug 1;21:11541. doi: 10.15766/mep_2374-8265.11541 (PMC12313986; doi:10.15766/mep_2374-8265.11541)
Supplement: Supplementary file 1 — Simulation Case.docxPatient HPI, Labs, and Imaging.pptxPre- and Postsimulation Surveys.docxFaculty Guide.docxDebriefing.pptxCritical Action Checklist.docx [file mep_2374-8265.11541-s001.zip › E. Debriefing.pptx]

## Slide 1
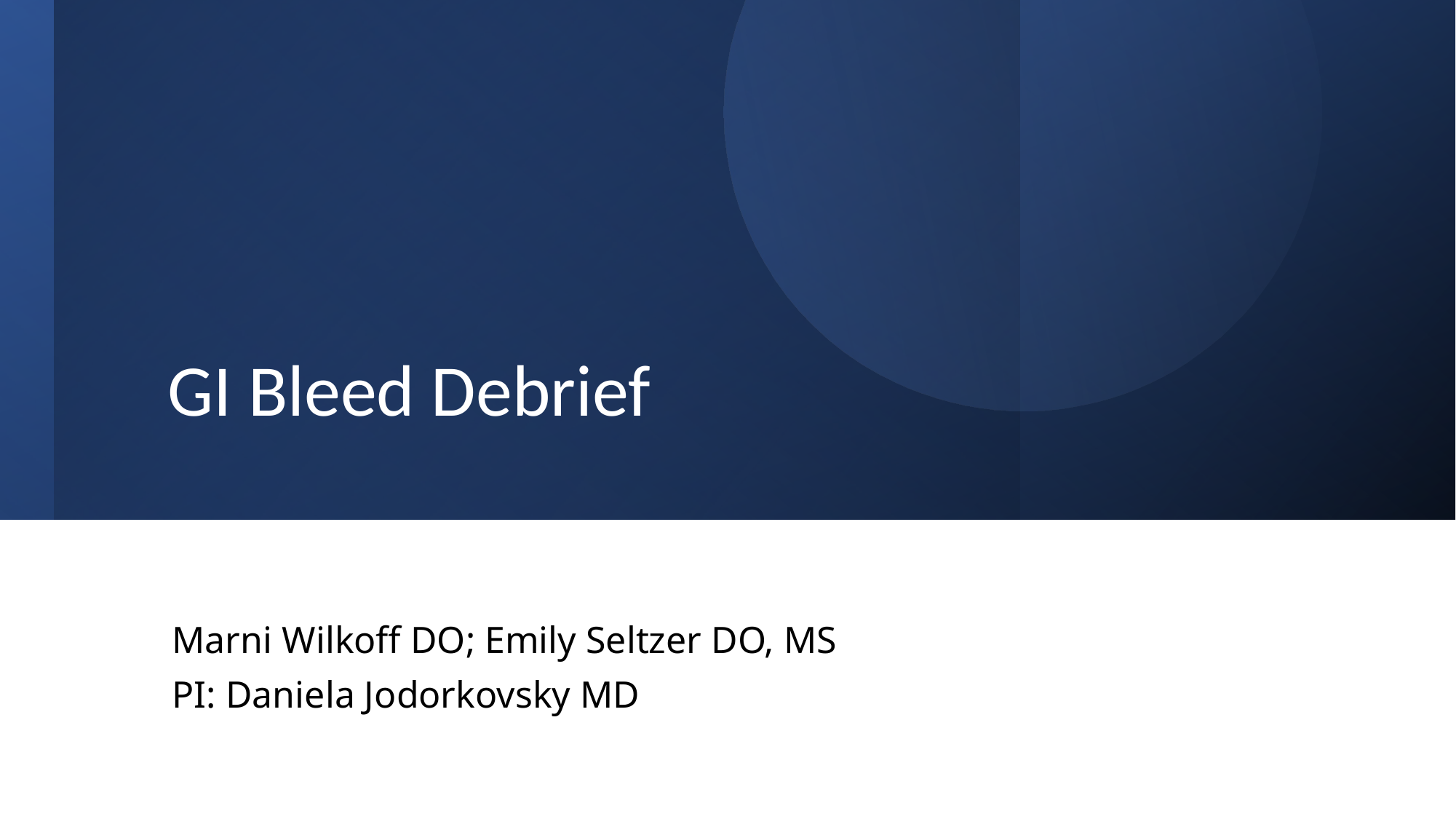

# GI Bleed Debrief
Marni Wilkoff DO; Emily Seltzer DO, MS
PI: Daniela Jodorkovsky MD

## Slide 2
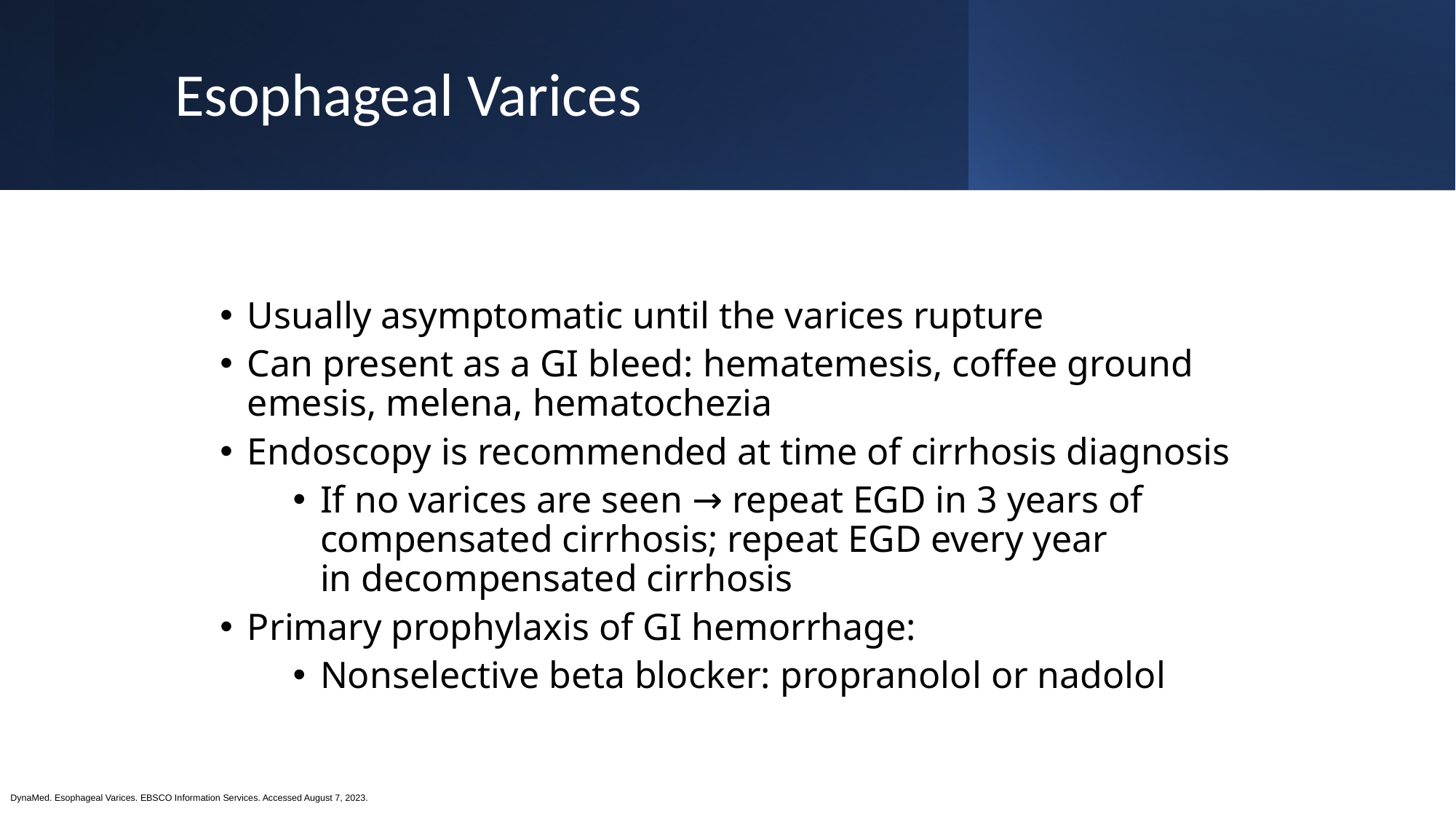

# Esophageal Varices
Usually asymptomatic until the varices rupture
Can present as a GI bleed: hematemesis, coffee ground emesis, melena, hematochezia
Endoscopy is recommended at time of cirrhosis diagnosis
If no varices are seen → repeat EGD in 3 years of compensated cirrhosis; repeat EGD every year in decompensated cirrhosis
Primary prophylaxis of GI hemorrhage:
Nonselective beta blocker: propranolol or nadolol
DynaMed. Esophageal Varices. EBSCO Information Services. Accessed August 7, 2023.

## Slide 3
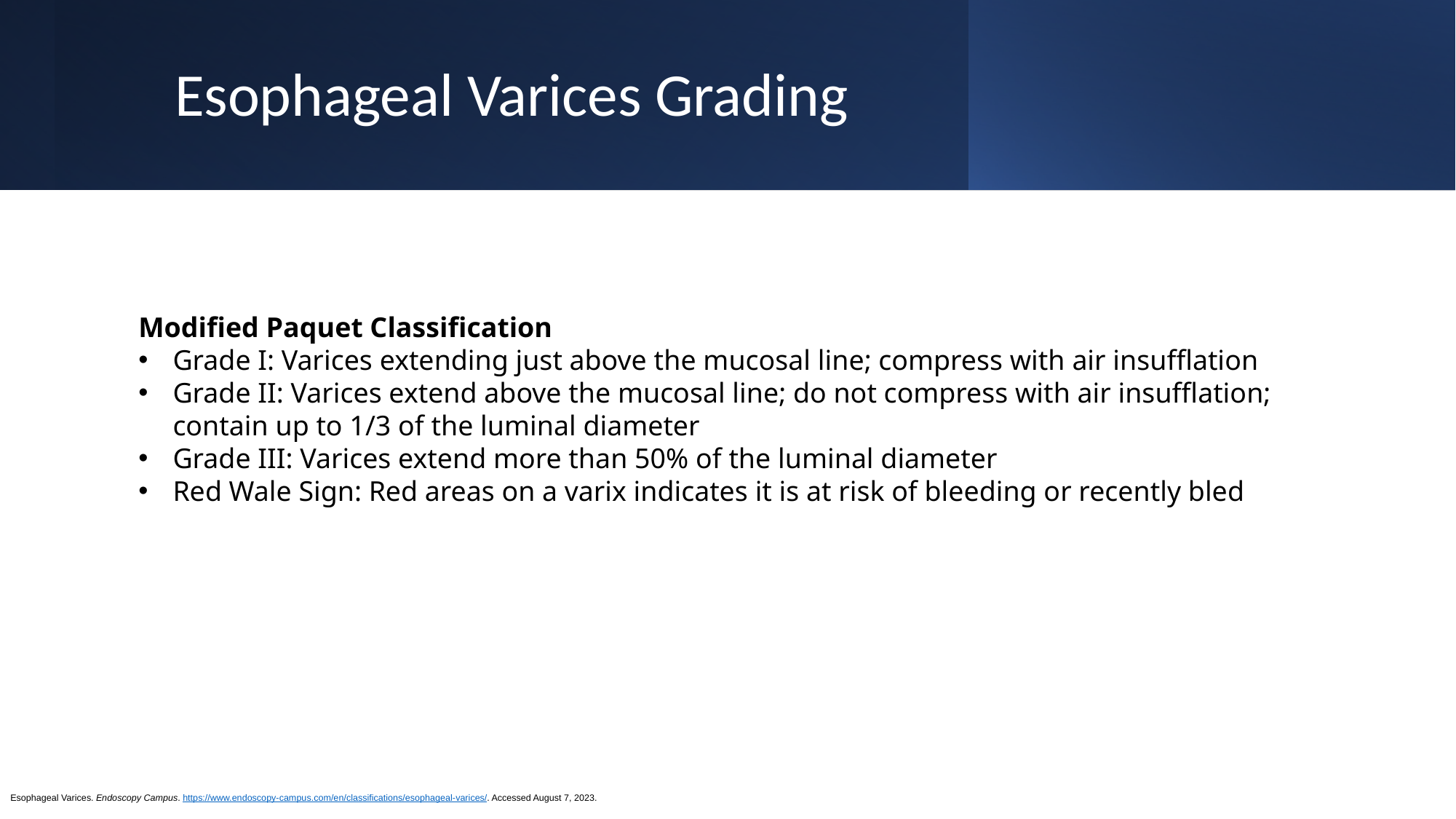

# Esophageal Varices Grading
Modified Paquet Classification
Grade I: Varices extending just above the mucosal line; compress with air insufflation
Grade II: Varices extend above the mucosal line; do not compress with air insufflation; contain up to 1/3 of the luminal diameter
Grade III: Varices extend more than 50% of the luminal diameter
Red Wale Sign: Red areas on a varix indicates it is at risk of bleeding or recently bled
Esophageal Varices. Endoscopy Campus. https://www.endoscopy-campus.com/en/classifications/esophageal-varices/. Accessed August 7, 2023.

## Slide 4
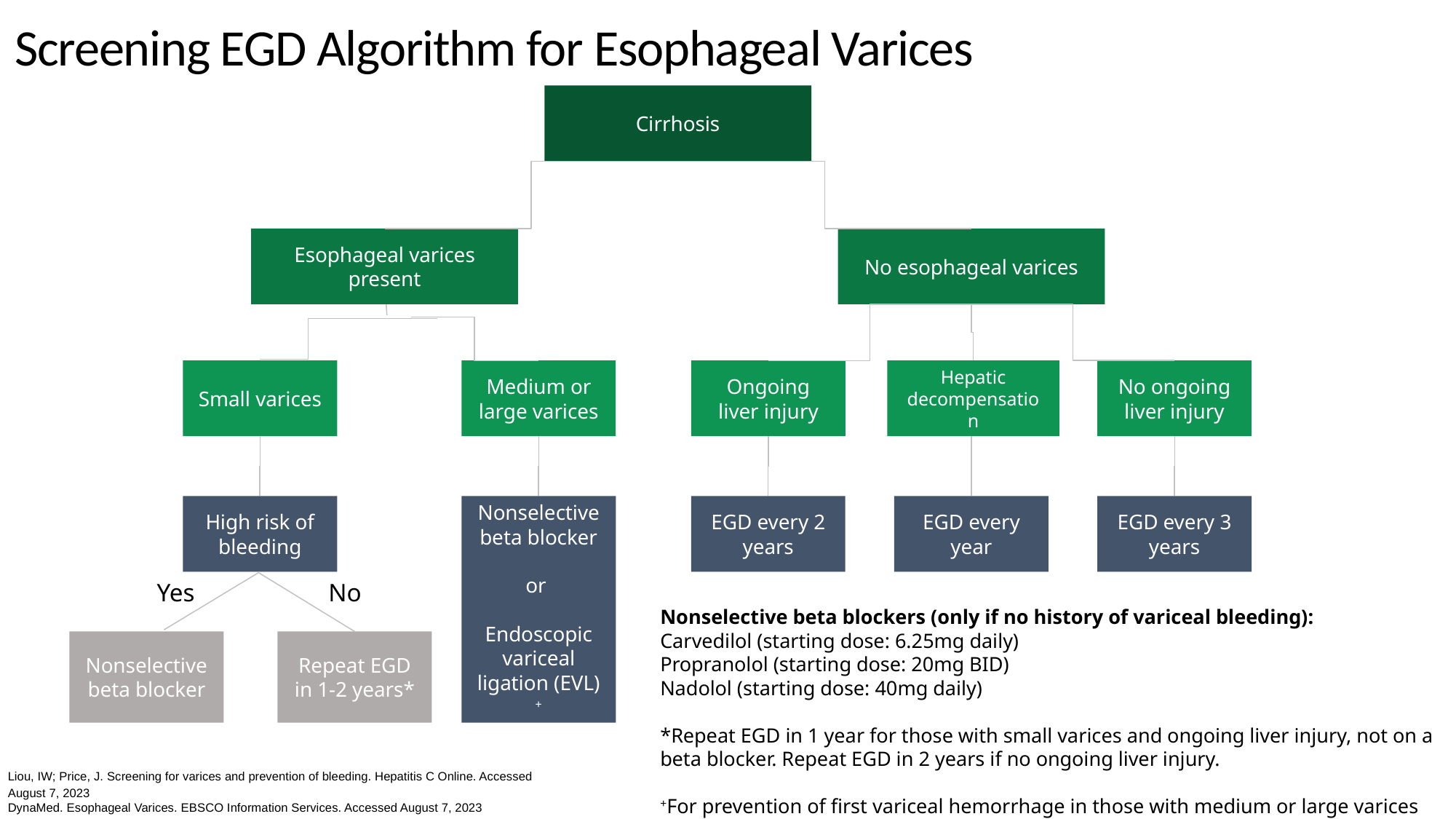

Screening EGD Algorithm for Esophageal Varices
Cirrhosis
Esophageal varices present
No esophageal varices
Small varices
Ongoing liver injury
No ongoing liver injury
Medium or large varices
Hepatic decompensation
High risk of bleeding
EGD every 2 years
EGD every 3 years
Nonselective beta blocker
or
Endoscopic variceal ligation (EVL)+
EGD every year
Yes
No
Nonselective beta blockers (only if no history of variceal bleeding):
Carvedilol (starting dose: 6.25mg daily)
Propranolol (starting dose: 20mg BID)
Nadolol (starting dose: 40mg daily)
*Repeat EGD in 1 year for those with small varices and ongoing liver injury, not on a beta blocker. Repeat EGD in 2 years if no ongoing liver injury.
+For prevention of first variceal hemorrhage in those with medium or large varices
Repeat EGD in 1-2 years*
Nonselective beta blocker
Liou, IW; Price, J. Screening for varices and prevention of bleeding. Hepatitis C Online. Accessed August 7, 2023
DynaMed. Esophageal Varices. EBSCO Information Services. Accessed August 7, 2023

## Slide 5
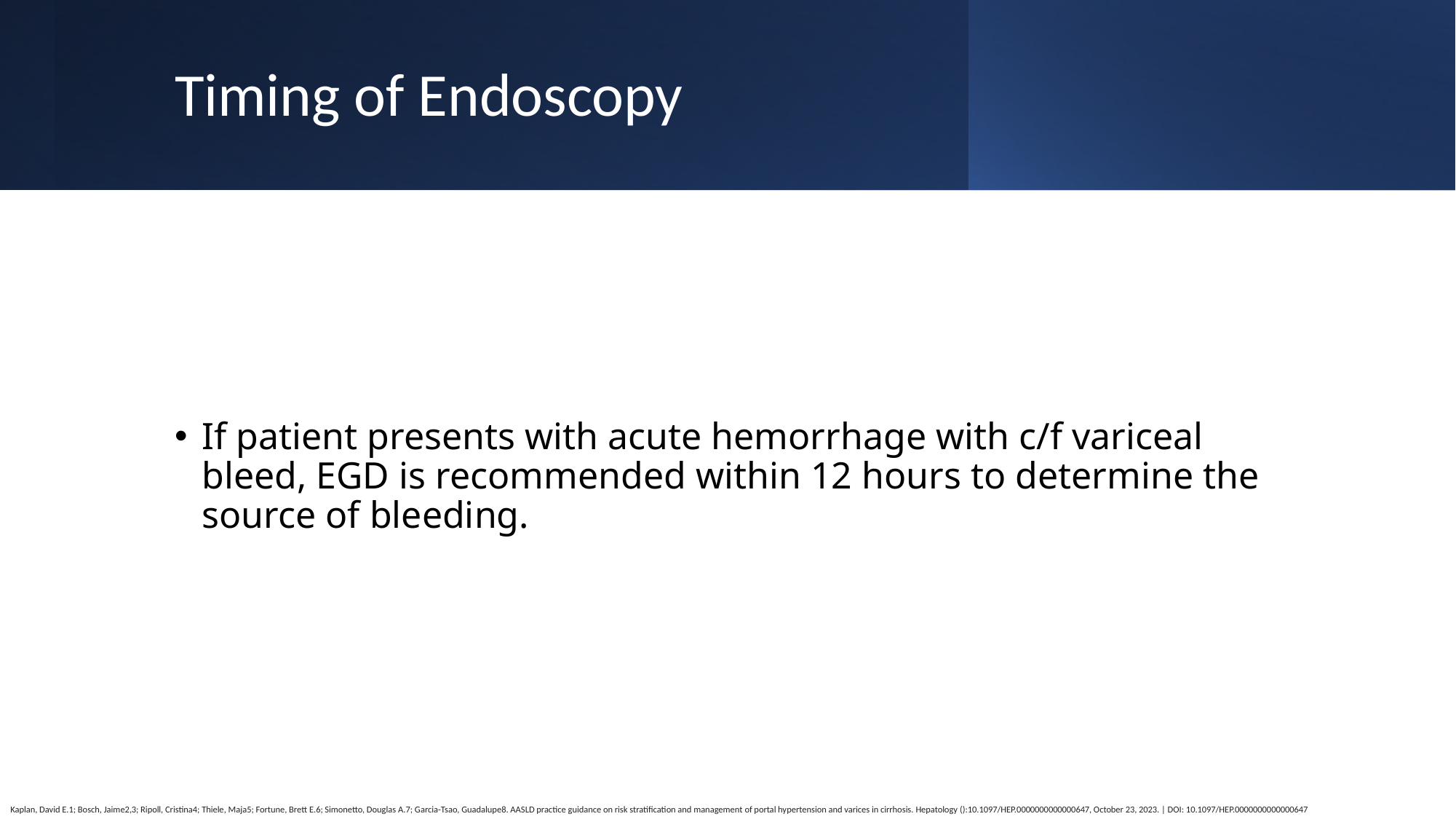

# Timing of Endoscopy
If patient presents with acute hemorrhage with c/f variceal bleed, EGD is recommended within 12 hours to determine the source of bleeding.
Kaplan, David E.1; Bosch, Jaime2,3; Ripoll, Cristina4; Thiele, Maja5; Fortune, Brett E.6; Simonetto, Douglas A.7; Garcia-Tsao, Guadalupe8. AASLD practice guidance on risk stratification and management of portal hypertension and varices in cirrhosis. Hepatology ():10.1097/HEP.0000000000000647, October 23, 2023. | DOI: 10.1097/HEP.0000000000000647

## Slide 6
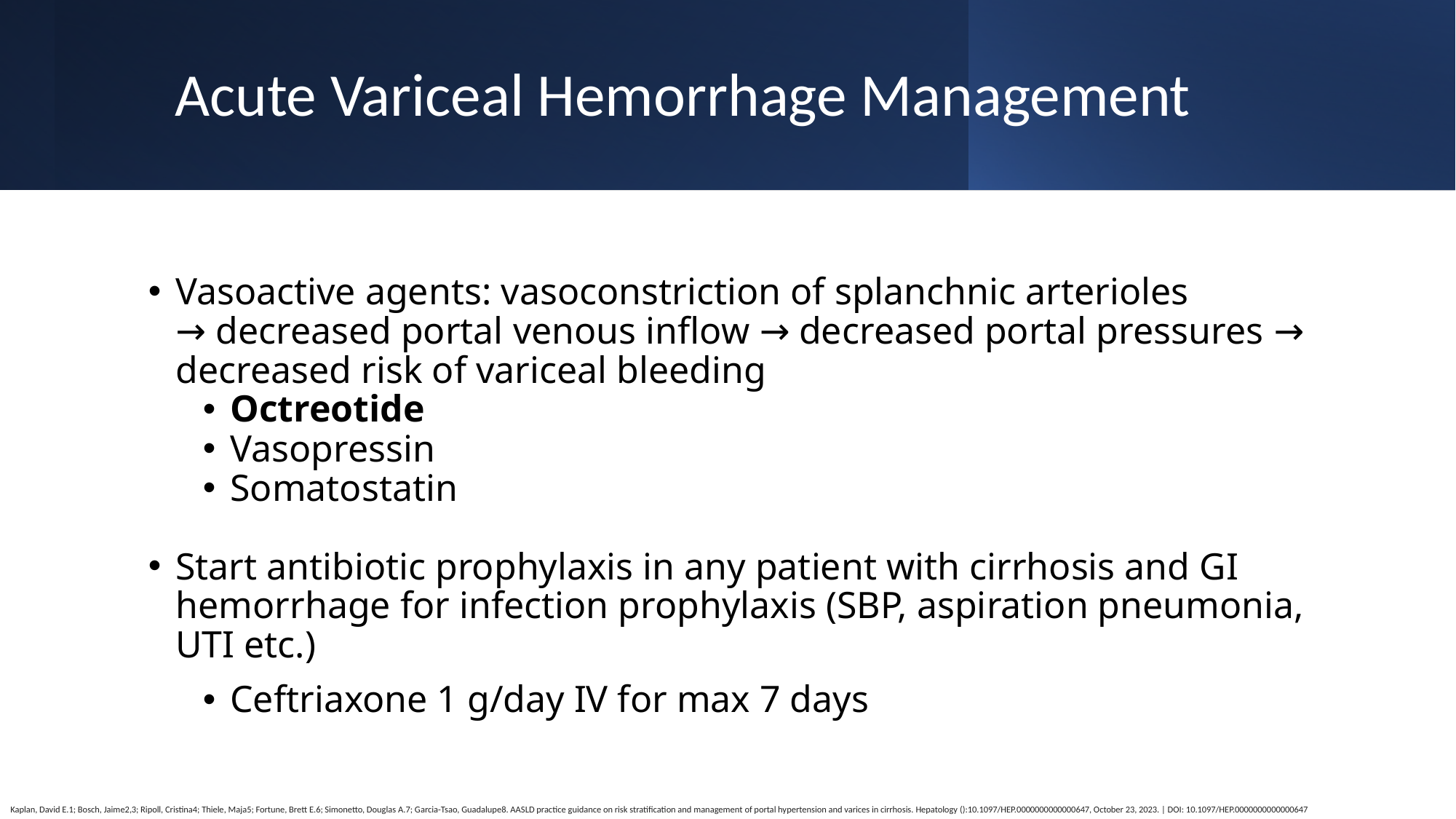

# Acute Variceal Hemorrhage Management
Vasoactive agents: vasoconstriction of splanchnic arterioles → decreased portal venous inflow → decreased portal pressures → decreased risk of variceal bleeding
Octreotide
Vasopressin
Somatostatin
Start antibiotic prophylaxis in any patient with cirrhosis and GI hemorrhage for infection prophylaxis (SBP, aspiration pneumonia, UTI etc.)
Ceftriaxone 1 g/day IV for max 7 days
Kaplan, David E.1; Bosch, Jaime2,3; Ripoll, Cristina4; Thiele, Maja5; Fortune, Brett E.6; Simonetto, Douglas A.7; Garcia-Tsao, Guadalupe8. AASLD practice guidance on risk stratification and management of portal hypertension and varices in cirrhosis. Hepatology ():10.1097/HEP.0000000000000647, October 23, 2023. | DOI: 10.1097/HEP.0000000000000647

## Slide 7
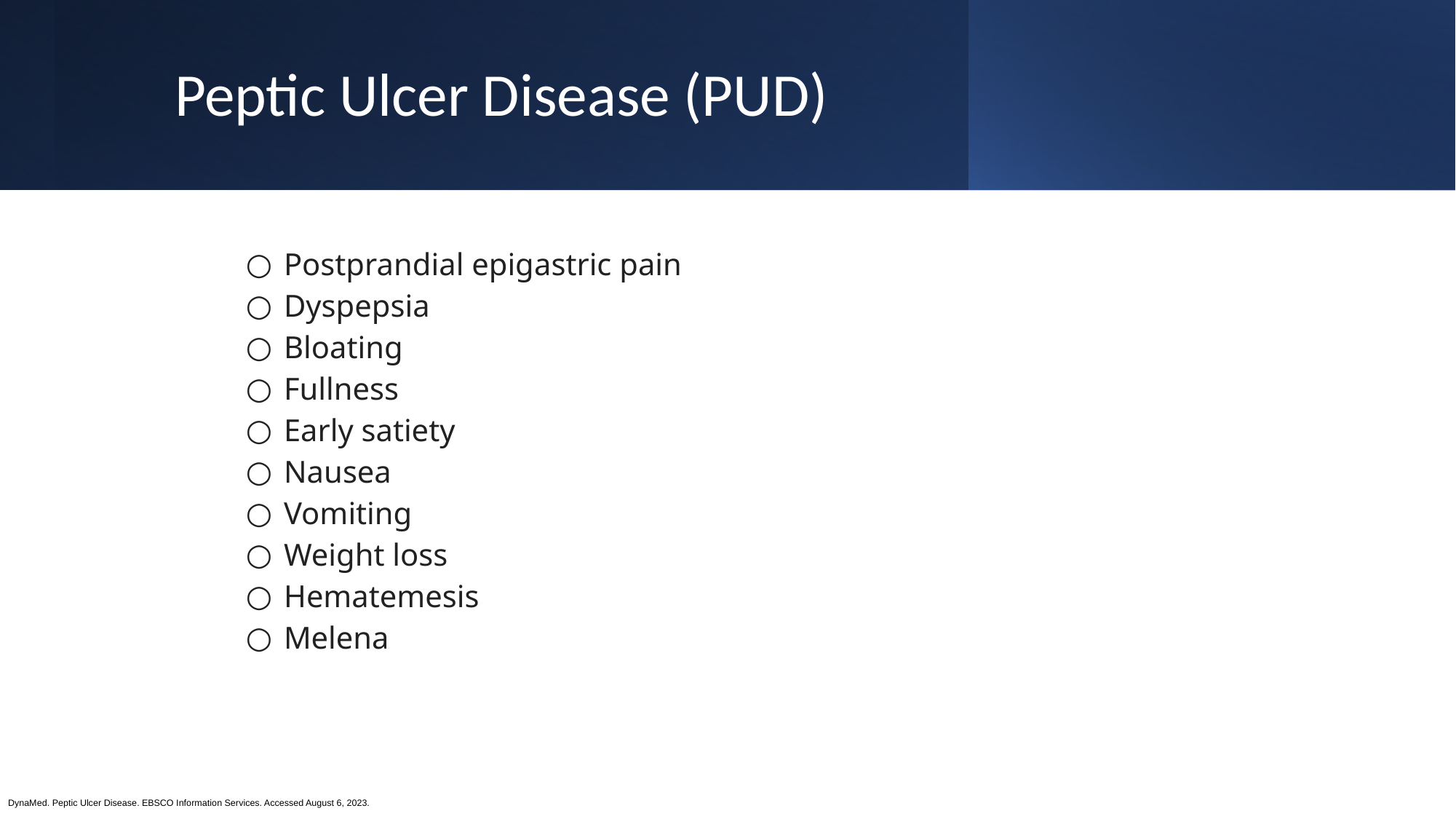

# Peptic Ulcer Disease (PUD)
Postprandial epigastric pain
Dyspepsia
Bloating
Fullness
Early satiety
Nausea
Vomiting
Weight loss
Hematemesis
Melena
DynaMed. Peptic Ulcer Disease. EBSCO Information Services. Accessed August 6, 2023.

## Slide 8
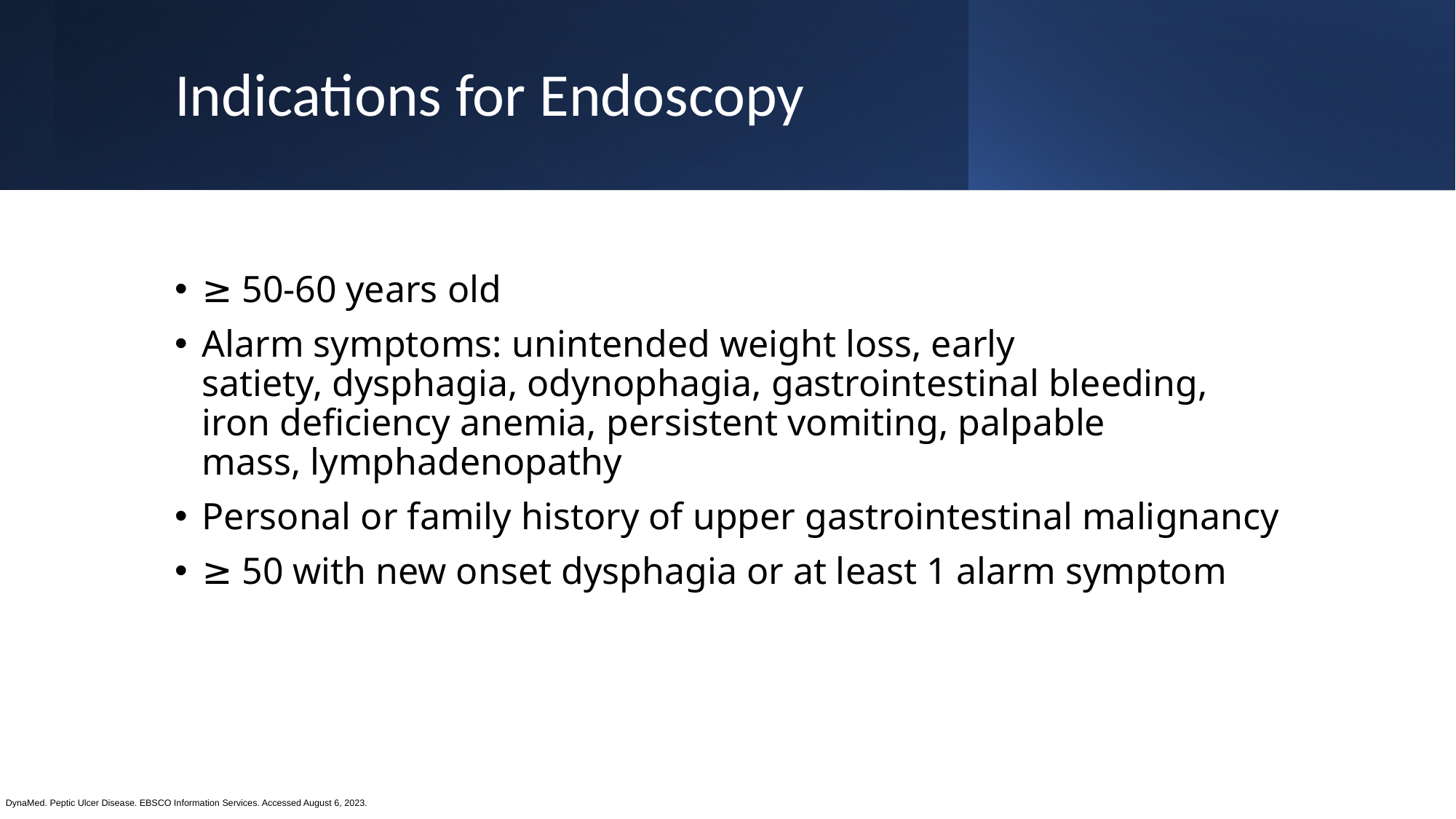

# Indications for Endoscopy
≥ 50-60 years old
Alarm symptoms: unintended weight loss, early satiety, dysphagia, odynophagia, gastrointestinal bleeding, iron deficiency anemia, persistent vomiting, palpable mass, lymphadenopathy
Personal or family history of upper gastrointestinal malignancy
≥ 50 with new onset dysphagia or at least 1 alarm symptom
DynaMed. Peptic Ulcer Disease. EBSCO Information Services. Accessed August 6, 2023.

## Slide 9
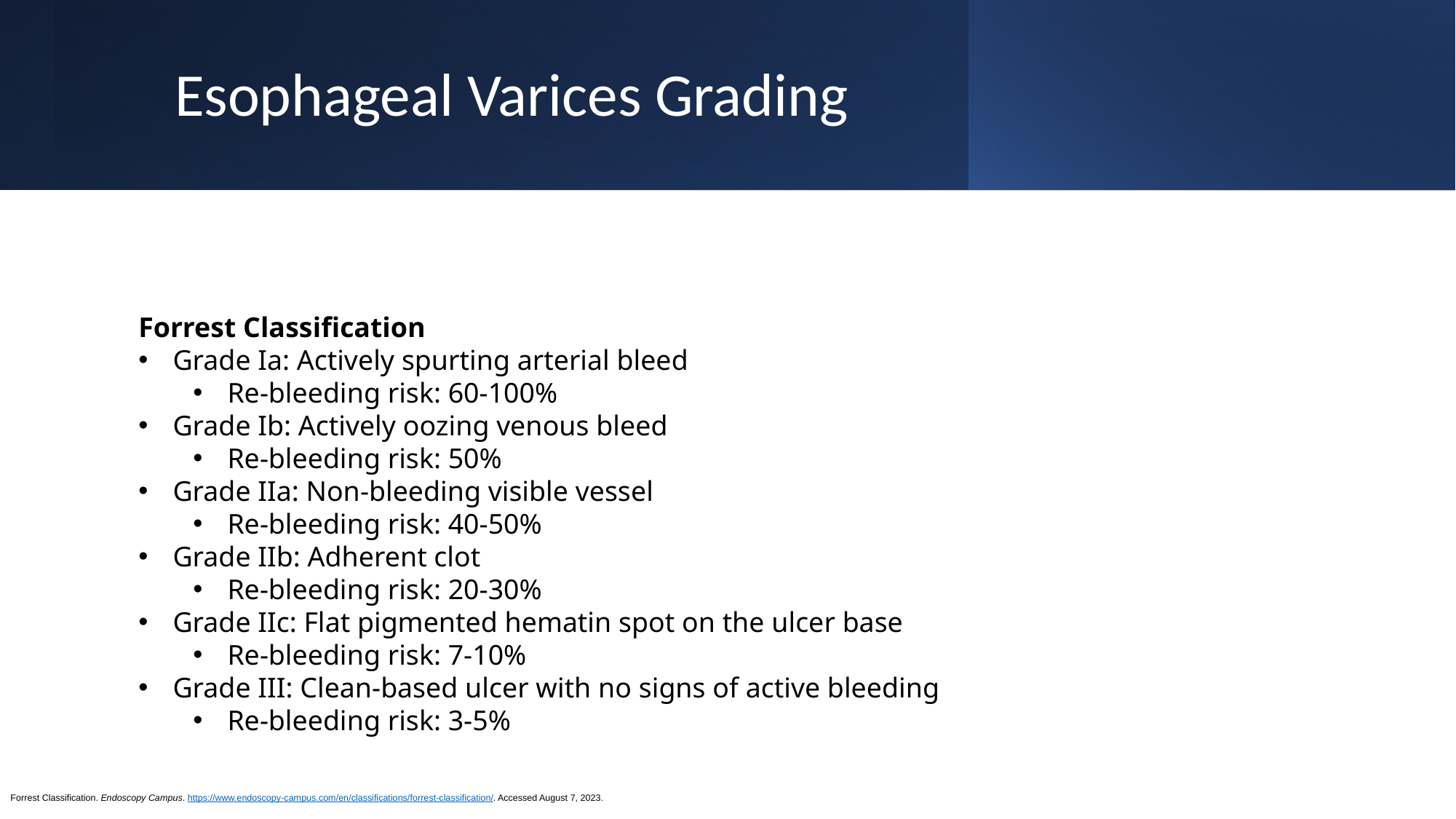

# Esophageal Varices Grading
Forrest Classification
Grade Ia: Actively spurting arterial bleed
Re-bleeding risk: 60-100%
Grade Ib: Actively oozing venous bleed
Re-bleeding risk: 50%
Grade IIa: Non-bleeding visible vessel
Re-bleeding risk: 40-50%
Grade IIb: Adherent clot
Re-bleeding risk: 20-30%
Grade IIc: Flat pigmented hematin spot on the ulcer base
Re-bleeding risk: 7-10%
Grade III: Clean-based ulcer with no signs of active bleeding
Re-bleeding risk: 3-5%
Forrest Classification. Endoscopy Campus. https://www.endoscopy-campus.com/en/classifications/forrest-classification/. Accessed August 7, 2023.

## Slide 10
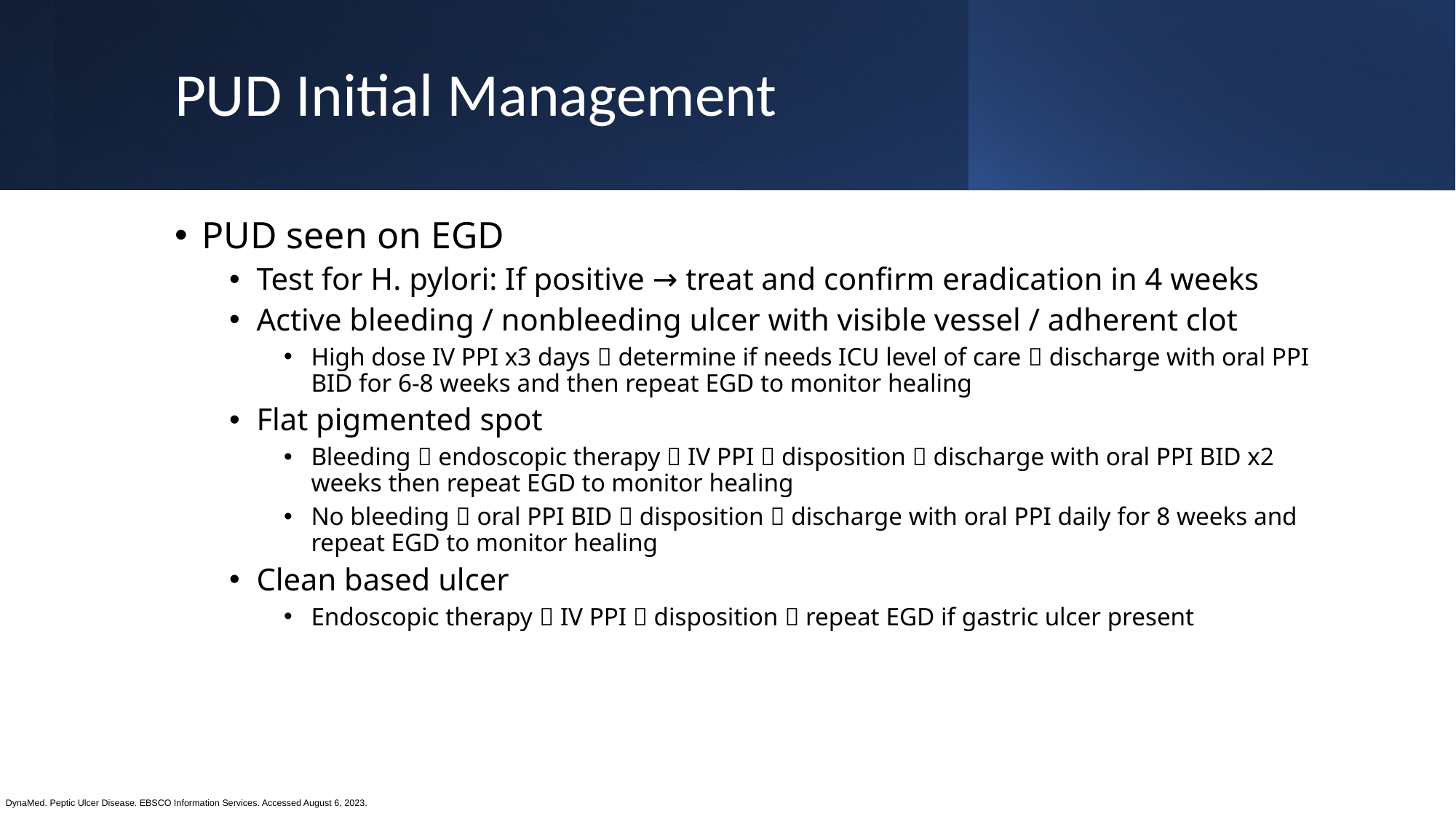

# PUD Initial Management
PUD seen on EGD
Test for H. pylori: If positive → treat and confirm eradication in 4 weeks
Active bleeding / nonbleeding ulcer with visible vessel / adherent clot
High dose IV PPI x3 days  determine if needs ICU level of care  discharge with oral PPI BID for 6-8 weeks and then repeat EGD to monitor healing
Flat pigmented spot
Bleeding  endoscopic therapy  IV PPI  disposition  discharge with oral PPI BID x2 weeks then repeat EGD to monitor healing
No bleeding  oral PPI BID  disposition  discharge with oral PPI daily for 8 weeks and repeat EGD to monitor healing
Clean based ulcer
Endoscopic therapy  IV PPI  disposition  repeat EGD if gastric ulcer present
DynaMed. Peptic Ulcer Disease. EBSCO Information Services. Accessed August 6, 2023.

## Slide 11
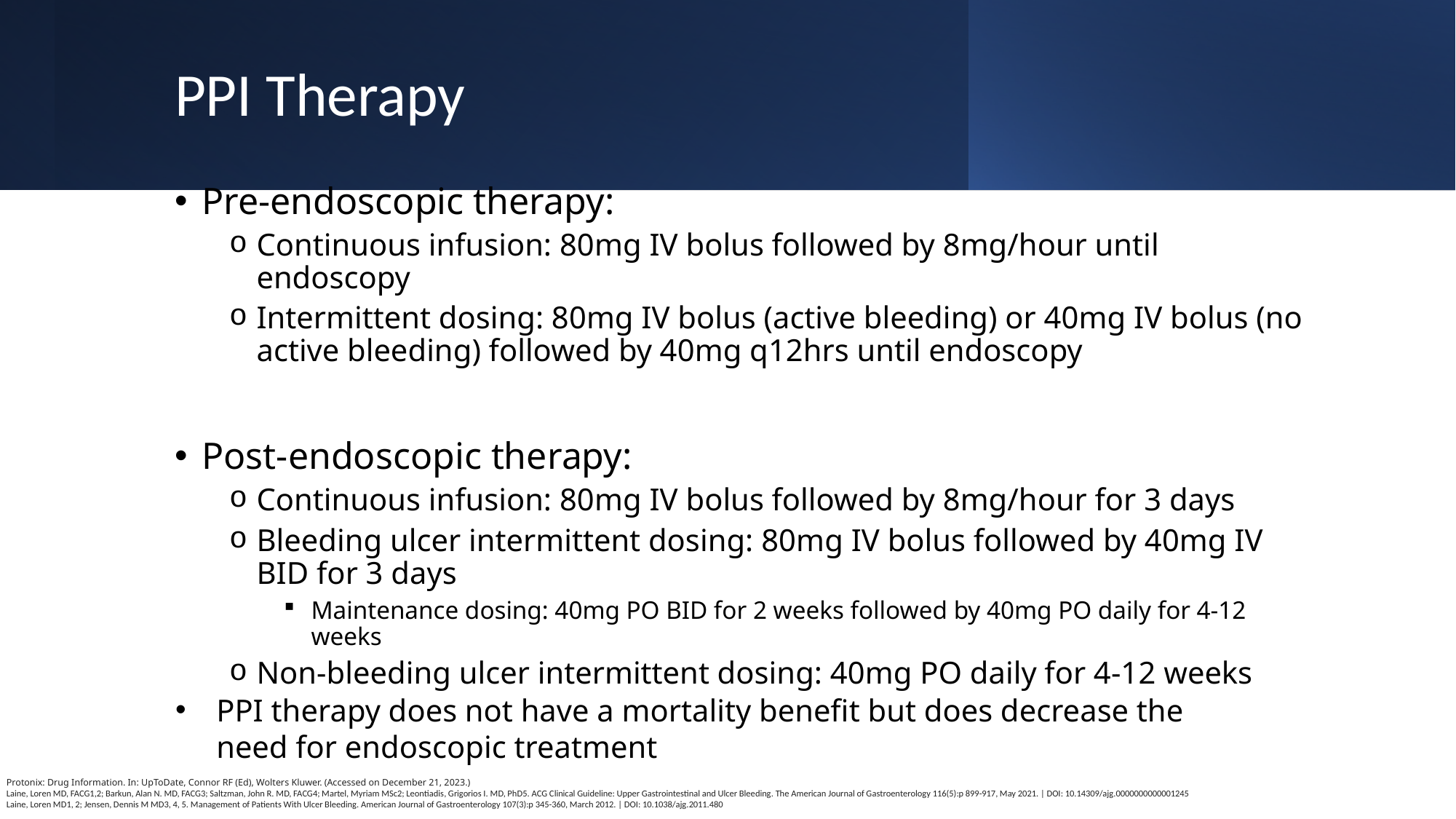

# PPI Therapy
Pre-endoscopic therapy:
Continuous infusion: 80mg IV bolus followed by 8mg/hour until endoscopy
Intermittent dosing: 80mg IV bolus (active bleeding) or 40mg IV bolus (no active bleeding) followed by 40mg q12hrs until endoscopy
Post-endoscopic therapy:
Continuous infusion: 80mg IV bolus followed by 8mg/hour for 3 days
Bleeding ulcer intermittent dosing: 80mg IV bolus followed by 40mg IV BID for 3 days
Maintenance dosing: 40mg PO BID for 2 weeks followed by 40mg PO daily for 4-12 weeks
Non-bleeding ulcer intermittent dosing: 40mg PO daily for 4-12 weeks
PPI therapy does not have a mortality benefit but does decrease the need for endoscopic treatment
Protonix: Drug Information. In: UpToDate, Connor RF (Ed), Wolters Kluwer. (Accessed on December 21, 2023.)
Laine, Loren MD, FACG1,2; Barkun, Alan N. MD, FACG3; Saltzman, John R. MD, FACG4; Martel, Myriam MSc2; Leontiadis, Grigorios I. MD, PhD5. ACG Clinical Guideline: Upper Gastrointestinal and Ulcer Bleeding. The American Journal of Gastroenterology 116(5):p 899-917, May 2021. | DOI: 10.14309/ajg.0000000000001245
Laine, Loren MD1, 2; Jensen, Dennis M MD3, 4, 5. Management of Patients With Ulcer Bleeding. American Journal of Gastroenterology 107(3):p 345-360, March 2012. | DOI: 10.1038/ajg.2011.480

## Slide 12
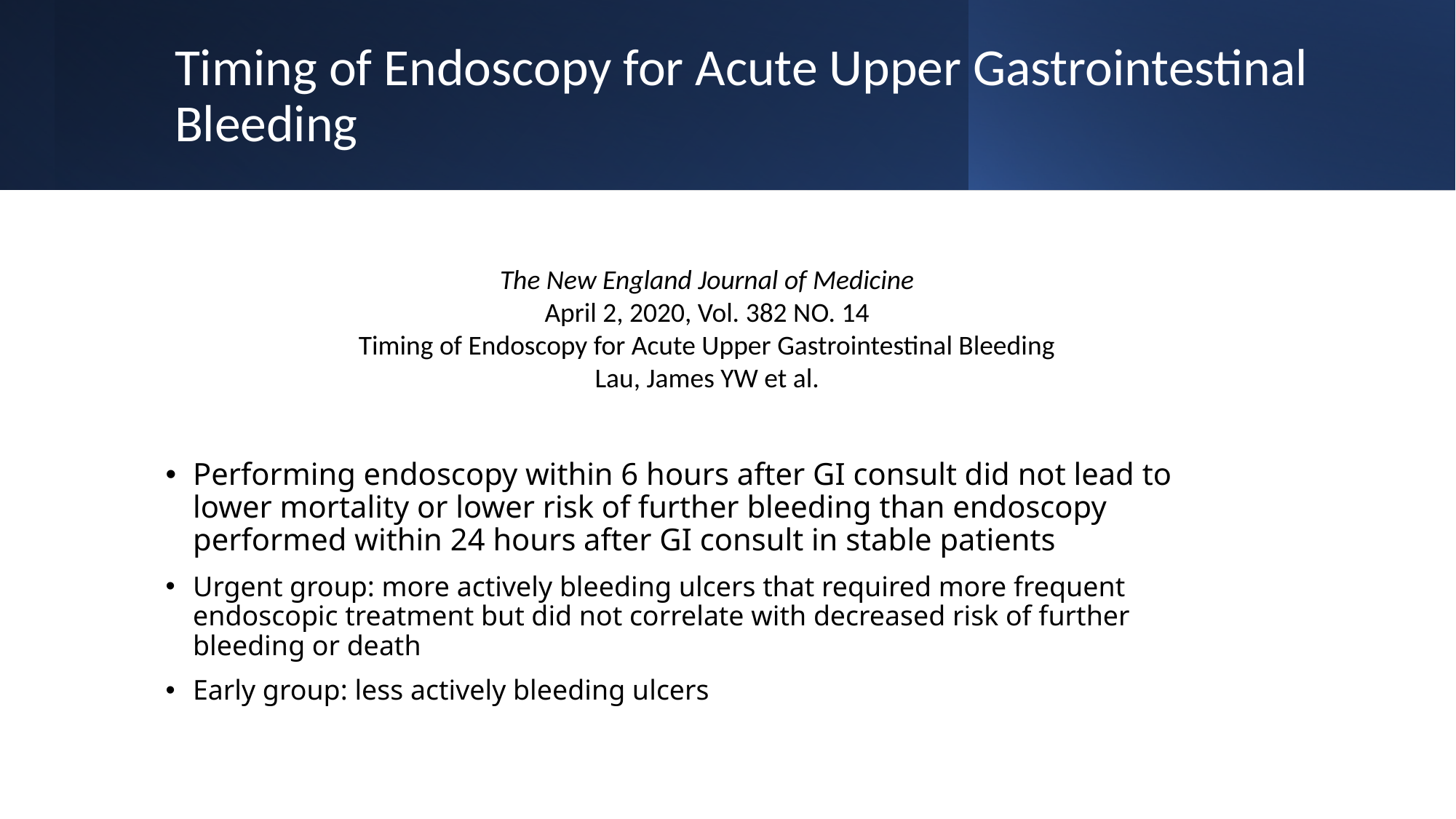

# Timing of Endoscopy for Acute Upper Gastrointestinal Bleeding
The New England Journal of Medicine
April 2, 2020, Vol. 382 NO. 14
Timing of Endoscopy for Acute Upper Gastrointestinal Bleeding
Lau, James YW et al.
Performing endoscopy within 6 hours after GI consult did not lead to lower mortality or lower risk of further bleeding than endoscopy performed within 24 hours after GI consult in stable patients
Urgent group: more actively bleeding ulcers that required more frequent endoscopic treatment but did not correlate with decreased risk of further bleeding or death
Early group: less actively bleeding ulcers

## Slide 13
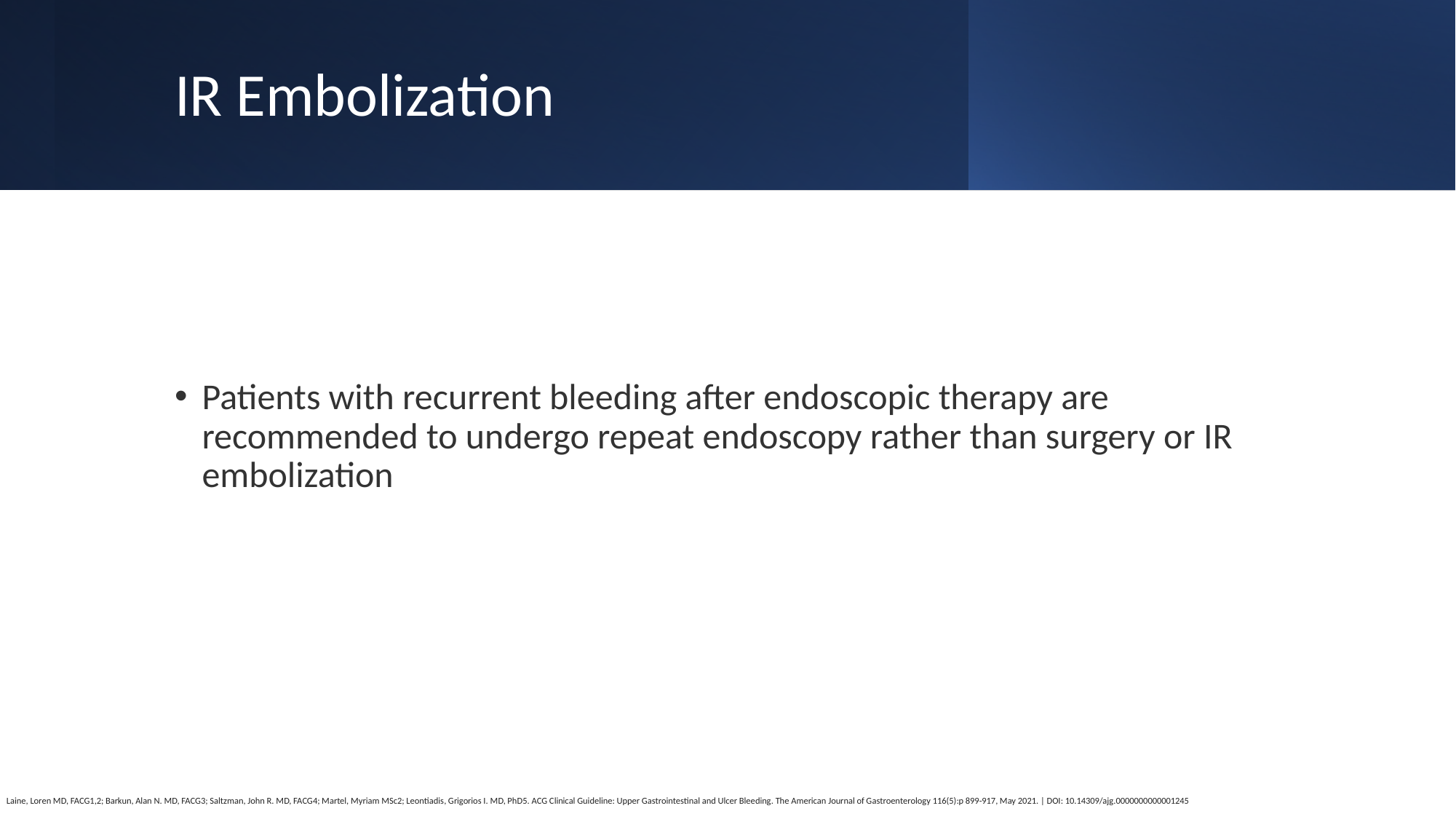

# IR Embolization
Patients with recurrent bleeding after endoscopic therapy are recommended to undergo repeat endoscopy rather than surgery or IR embolization
Laine, Loren MD, FACG1,2; Barkun, Alan N. MD, FACG3; Saltzman, John R. MD, FACG4; Martel, Myriam MSc2; Leontiadis, Grigorios I. MD, PhD5. ACG Clinical Guideline: Upper Gastrointestinal and Ulcer Bleeding. The American Journal of Gastroenterology 116(5):p 899-917, May 2021. | DOI: 10.14309/ajg.0000000000001245

## Slide 14
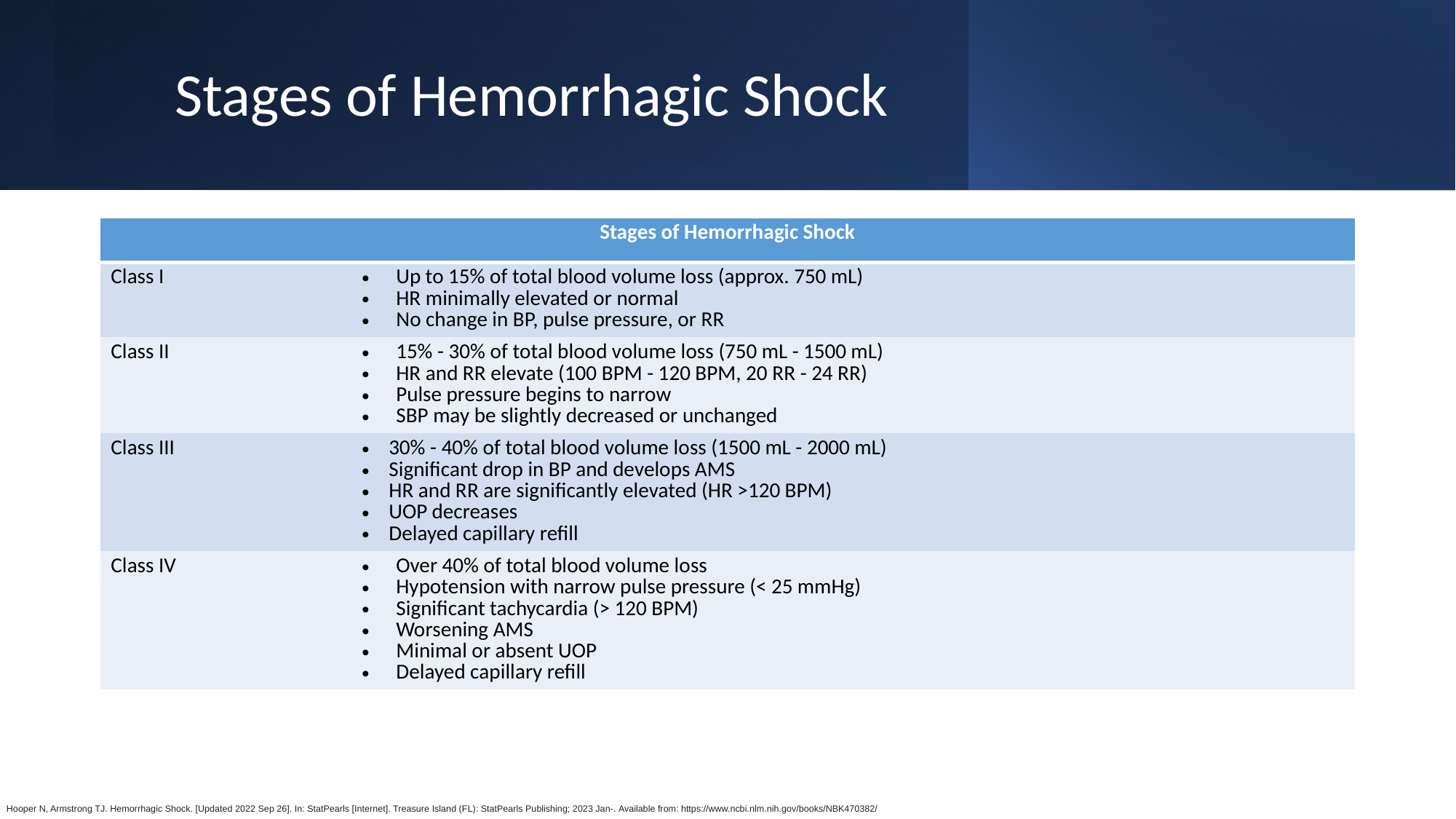

# Stages of Hemorrhagic Shock
| Stages of Hemorrhagic Shock | |
| --- | --- |
| Class I | Up to 15% of total blood volume loss (approx. 750 mL)  HR minimally elevated or normal No change in BP, pulse pressure, or RR |
| Class II | 15% - 30% of total blood volume loss (750 mL - 1500 mL) HR and RR elevate (100 BPM - 120 BPM, 20 RR - 24 RR) Pulse pressure begins to narrow SBP may be slightly decreased or unchanged |
| Class III | 30% - 40% of total blood volume loss (1500 mL - 2000 mL)  Significant drop in BP and develops AMS HR and RR are significantly elevated (HR >120 BPM) UOP decreases Delayed capillary refill |
| Class IV | Over 40% of total blood volume loss Hypotension with narrow pulse pressure (< 25 mmHg) Significant tachycardia (> 120 BPM) Worsening AMS Minimal or absent UOP Delayed capillary refill |
Hooper N, Armstrong TJ. Hemorrhagic Shock. [Updated 2022 Sep 26]. In: StatPearls [Internet]. Treasure Island (FL): StatPearls Publishing; 2023 Jan-. Available from: https://www.ncbi.nlm.nih.gov/books/NBK470382/

## Slide 15
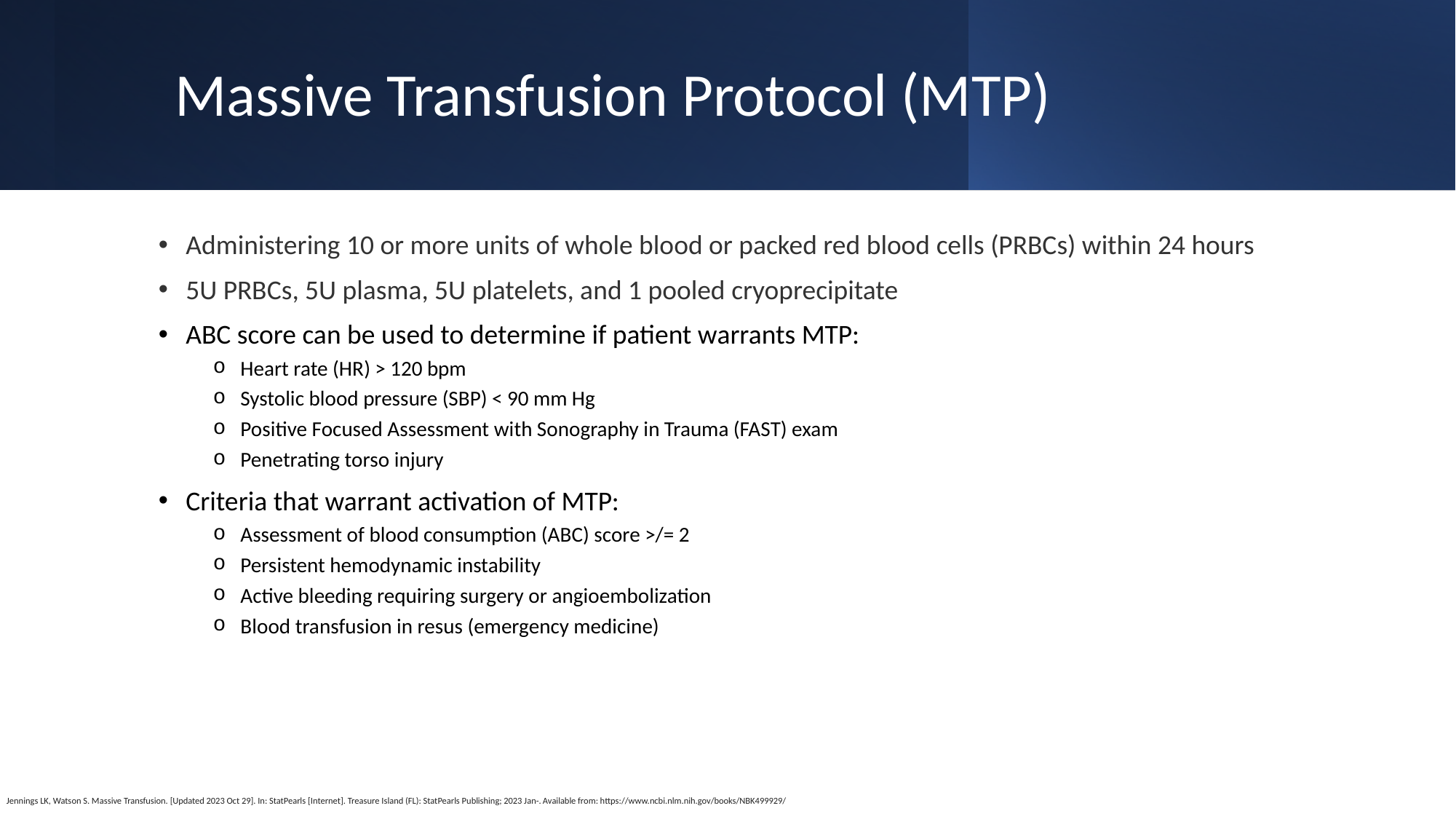

# Massive Transfusion Protocol (MTP)
Administering 10 or more units of whole blood or packed red blood cells (PRBCs) within 24 hours
5U PRBCs, 5U plasma, 5U platelets, and 1 pooled cryoprecipitate
ABC score can be used to determine if patient warrants MTP:
Heart rate (HR) > 120 bpm
Systolic blood pressure (SBP) < 90 mm Hg
Positive Focused Assessment with Sonography in Trauma (FAST) exam
Penetrating torso injury
Criteria that warrant activation of MTP:
Assessment of blood consumption (ABC) score >/= 2
Persistent hemodynamic instability
Active bleeding requiring surgery or angioembolization
Blood transfusion in resus (emergency medicine)
Jennings LK, Watson S. Massive Transfusion. [Updated 2023 Oct 29]. In: StatPearls [Internet]. Treasure Island (FL): StatPearls Publishing; 2023 Jan-. Available from: https://www.ncbi.nlm.nih.gov/books/NBK499929/

## Slide 16
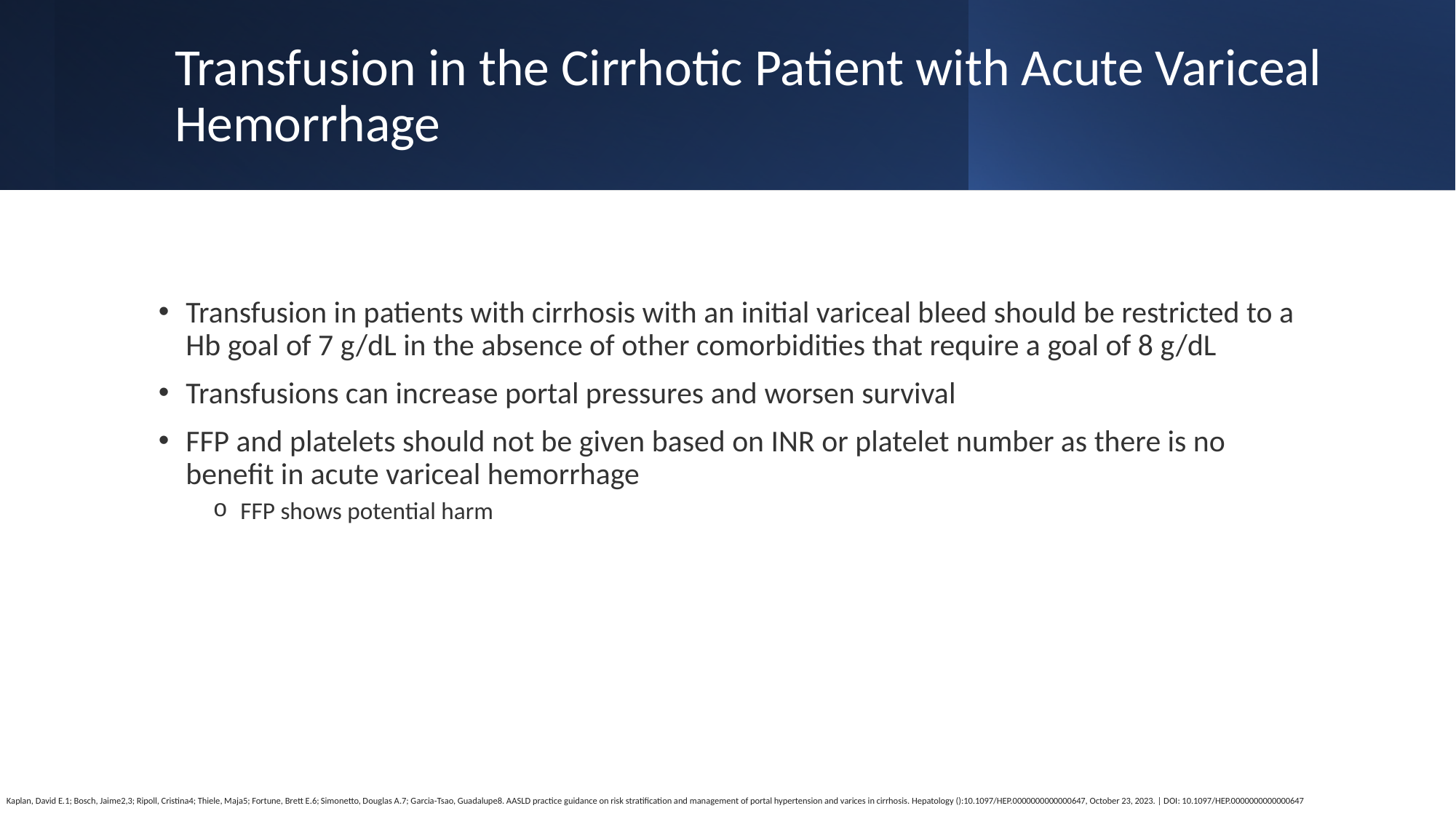

# Transfusion in the Cirrhotic Patient with Acute Variceal Hemorrhage
Transfusion in patients with cirrhosis with an initial variceal bleed should be restricted to a Hb goal of 7 g/dL in the absence of other comorbidities that require a goal of 8 g/dL
Transfusions can increase portal pressures and worsen survival
FFP and platelets should not be given based on INR or platelet number as there is no benefit in acute variceal hemorrhage
FFP shows potential harm
Kaplan, David E.1; Bosch, Jaime2,3; Ripoll, Cristina4; Thiele, Maja5; Fortune, Brett E.6; Simonetto, Douglas A.7; Garcia-Tsao, Guadalupe8. AASLD practice guidance on risk stratification and management of portal hypertension and varices in cirrhosis. Hepatology ():10.1097/HEP.0000000000000647, October 23, 2023. | DOI: 10.1097/HEP.0000000000000647

## Slide 17
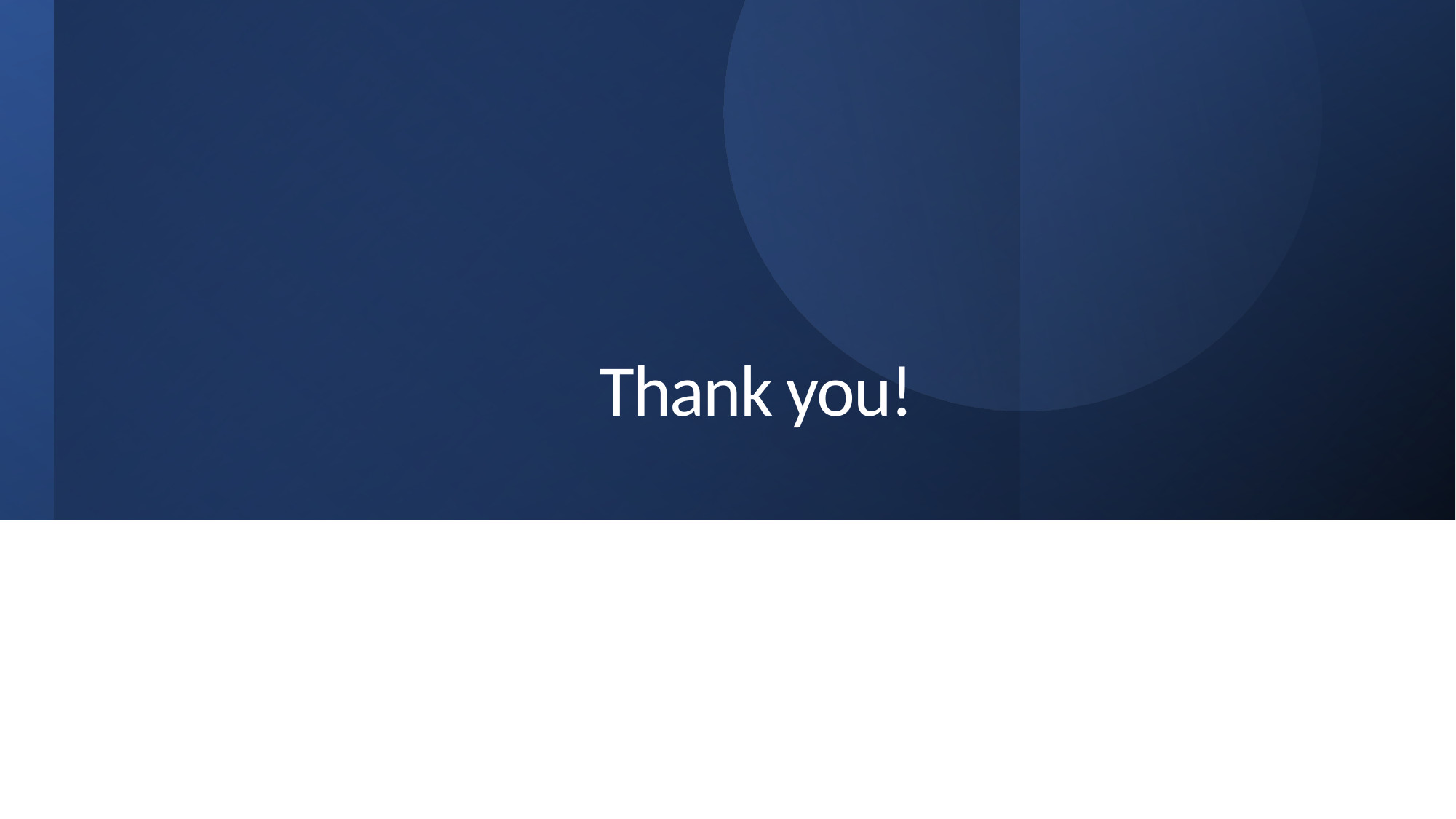

# Thank you!
