# Supplementary material for: Acute Upper Gastrointestinal Bleeding: A Hands-On Simulation Case for Internal Medicine Residents Improves Knowledge and Confidence
Source: MedEdPORTAL. 2025 Aug 1;21:11541. doi: 10.15766/mep_2374-8265.11541 (PMC12313986; doi:10.15766/mep_2374-8265.11541)
Supplement: Supplementary file 1 — Simulation Case.docxPatient HPI, Labs, and Imaging.pptxPre- and Postsimulation Surveys.docxFaculty Guide.docxDebriefing.pptxCritical Action Checklist.docx [file mep_2374-8265.11541-s001.zip › F. Critical Action Checklist.docx]

**Appendix F: Critical Actions Checklist**

**Critical Action Checklist**

| **Expected Learner Actions** | **Yes** | **No** |
| --- | --- | --- |
| Assess vital signs |  |  |
| Perform physical examination |  |  |
| Find melena during physical exam |  |  |
| Ask nurse if patient had hematemesis |  |  |
| Obtain additional IV access or central access |  |  |
| Ask for labs (ex: CBC, CMP, coagulation studies, fibrinogen, type and screen, blood gas with lactate) |  |  |
| Order IV Octreotide 50mcg bolus followed by 50mcg/hr drip |  |  |
| Order IV Protonix 80mg bolus followed by 8mg/hr drip or intermittent dosing |  |  |
| Order Ceftriaxone 1g daily |  |  |
| Intubate the patient for airway protection |  |  |
| Consult the ICU |  |  |
| Consult GI for endoscopy |  |  |
| Prescribe nonselective beta blocker and PO PPI upon discharge |  |  |
