# Supplementary material for: Acute Upper Gastrointestinal Bleeding: A Hands-On Simulation Case for Internal Medicine Residents Improves Knowledge and Confidence
Source: MedEdPORTAL. 2025 Aug 1;21:11541. doi: 10.15766/mep_2374-8265.11541 (PMC12313986; doi:10.15766/mep_2374-8265.11541)
Supplement: Supplementary file 1 — Simulation Case.docxPatient HPI, Labs, and Imaging.pptxPre- and Postsimulation Surveys.docxFaculty Guide.docxDebriefing.pptxCritical Action Checklist.docx [file mep_2374-8265.11541-s001.zip › A. Simulation Case.docx]

| **Appendix A: *MedEdPORTAL* Simulation Case Template**  **SIMULATION CASE TITLE:** Diagnosis and Management of Upper GI Bleeds  **AUTHORS:** Marni H Wilkoff DO, Emily S Seltzer DO, MS, Daniela Jodorkovsky MD | |
| --- | --- |
| **PATIENT NAME:** John Doe  **PATIENT AGE:** 45  **CHIEF COMPLAINT:** Abdominal swelling and pain | |
| **Brief narrative description of case** | Mr. Doe is a 45-year-old male with a past medical history of alcohol use disorder and chronic back pain who presents to the hospital for 1 week of abdominal swelling and pain. A rapid response was called 12 hours after admission for hematemesis associated with hemodynamic instability, requiring intubation, massive transfusion protocol and endoscopic intervention.  Learners are expected to recognize hemorrhagic shock secondary to peptic ulcer disease or esophageal varices causing an upper gastrointestinal bleed and perform diagnostic workup, resuscitation, and therapeutic measures. |
| **Primary Learning Objectives** | 1. Create a differential diagnosis for upper GI bleeds. 2. List the indications for massive transfusion protocol in an unstable upper GI bleed. 3. Learn how to resuscitate and manage the airway of an upper GI bleed. 4. Learn appropriate medical management of upper GI bleeds and timing of upper endoscopy. 5. Develop communication skills to talk to consultants regarding a critical patient. 6. Describe proper inpatient management of upper GI bleeds. |
| **Critical Actions** | 1. Assess vital sign abnormalities including hypotension, tachycardia and oxygenation. 2. Physical examination – evaluate indicators of hypoperfusion and level of hemorrhage. 3. Discuss need to activate massive transfusion protocol MTP. 4. Intubate patient for airway protection. 5. Obtain appropriate intravenous access – introducer or two large bore IVs. 6. Obtain necessary lab work – CBC, BMP, liver function tests, magnesium, coagulation studies, fibrinogen, type and cross, blood gas with lactate. 7. Give appropriate medications – octreotide bolus followed by infusion, protonix bolus followed by drip or intermittent dosing. 8. Call gastroenterology for endoscopy. 9. Create a differential diagnosis of upper GI bleed. 10. Patient stabilized – prescribe appropriate medications on discharge (nonselective beta blocker, proton pump inhibitor (PPI) twice daily). |
| **Learner Preparation** | No advanced knowledge is required. |

| **INITIAL PRESENTATION** | | | |
| --- | --- | --- | --- |
| **Initial vital signs** | On admission: T 98 F, BP 128/86, HR 76, RR 16, O2 98% | | |
| **Overall Appearance** | Lethargic patient, in acute distress, actively vomiting blood (estimated blood loss 500cc), hypotensive and tachycardic, cool to the touch**.** | | |
| **Actors and roles in the room at case start** | A covering nurse called the rapid response for tachycardia and worsening abdominal pain is present at the beginning and calls team into room. | | |
| **HPI** | Chief complaint: “Abdominal swelling and pain”  *Information given by patient when asked*:   - The patient is vomiting blood, feels fatigued, dizzy, and has severe abdominal pain (specifically epigastric if asked). - He never had this pain before. - He has been drinking 6 beers a day for 20 yrs and has chronic back pain for which he takes ibuprofen twice a day for the past 5 years.   *Information given by RN if asked*:   - Rapid response was called for tachycardia and worsening abdominal pain. - Patient was feeling well prior to the rapid response, though was continuing to endorse abdominal swelling and pain that worsened recently. | | |
| **Past Medical/Surgical History** | **Medications** | **Allergies** | **Family History** |
| Alcohol use disorder  Chronic back pain | Ibuprofen | None | None |
| **Physical Examination** | | | |
| **General** | Middle aged male in acute distress, occasionally moaning in pain, actively vomiting blood | | |
| **HEENT** | Normocephalic/atraumatic, pupils equally round and reactive to light and accommodation, extraocular movements intact, pale oral mucosa with dried blood in the oropharynx | | |
| **Neck** | Supple, no lymphadenopathy, no thyromegaly | | |
| **Lungs** | Clear to auscultation bilaterally | | |
| **Cardiovascular** | Tachycardic but regular rhythm, cool extremities, prolonged capillary refill | | |
| **Abdomen** | Diffuse abdominal tenderness to palpation, distension, + fluid wave (not able to be physically simulated, only noted in PowerPoint slides), + asterixis (not able to be physically simulated, only noted in PowerPoint slides)  Rectal: melena on digital rectal exam | | |
| **Neurological** | Lethargic but responsive, moving all 4 extremities spontaneously, no focal deficits | | |
| **Skin** | Pale, + spider angioma, + palmer erythema | | |

| **INSTRUCTOR NOTES - CHANGES AND CASE BRANCH POINTS** | | | |
| --- | --- | --- | --- |
| **Patient state/vitals** | **Patient Status / Clinical Updates** | **Learner Actions, Modifiers and Triggers to Move to the Next State** | |
| **0min: entry to the room**  BP 80/46, HR 130, RR 30, SpO2 90%, T 98F | **Patient**: lethargic, pale, blood in emesis basin | Expected Learner Actions  Place patient on monitor.  Obtain history and perform physical exam.  Obtain labs (CBC, BMP, LFTs, coagulation studies, venous blood gas with lactate, type and screen).  Obtain two 16g or 18g peripheral IV or place an introducer.  Order IV fluids. | Modifiers  If learners do not give fluids, the nurse notes patient is becoming more tachycardic (HR 140s).  If learners do obtain adequate IV access, the nurse mentions the current IV access isn’t adequate and asks what gauge IV the team would want or if they want central access.  Triggers  For progression to the next step: obtain adequate IV or central access; order IV fluids. |
| **5 minutes: Hemorrhagic shock and resuscitation**  BP 82/50, HR 132, RR 28, SpO2 90%, T 98F | **Patient**: fatigued, pale, vomits 500cc bright red blood  **Notable labs:**  Hemoglobin 6.4 (down from 9.3 on admission)  Platelet 82  VBG: pH 7.28, lactate 3.1  INR 2.8 | Expected Learner Actions:  Order 1 unit of packed red blood cells.  Order octreotide 50mcg bolus followed by 50mcg/hr drip and Protonix 80mg IV bolus followed by continuous or intermittent dosing.  Order IV ceftriaxone.  Consult GI for endoscopy (team must highlight the urgency of the consult as there is suspicion for a variceal bleed). | Modifiers  If the team doesn’t order packed red blood cells, the HR will increase to 140 and the systolic blood pressure will drop to 70.  When the team calls GI, they will ask the team to adequately resuscitate the patient as well as what medications they have given thus far.  If the team doesn’t order ceftriaxone, GI ask them to place the patient on spontaneous bacterial peritonitis (SBP) prophylaxis.  Triggers:  For progression to the next step: order packed red blood cells, order octreotide and IV PPI, consult GI. |
| **10 minutes: Intubation**  BP 82/50, HR 132, RR 28, SpO2 90%, T 98 | **Patient**: lethargic, pale | Expected Learner Actions:  Consult anesthesia for intubation.  Suction blood from patient’s mouth.  Order post-intubation chest Xray. | Modifiers  If the team does not call anesthesia for intubation, the nurse will say “it looks like the patient is having difficulty breathing.”  If proper suction is not performed prior to intubation, the patient will have multiple episodes of emesis. Anesthesia will say it is difficult to intubate the patient who will become more hypoxic.  Triggers  For progression to the next step: successful intubation. |
| **15 minutes: Transfer to ICU and endoscopy**  BP 98/60, HR 110, RR 18, SpO2 97%, T 98 | **Patient:** intubated, sedated  Chest Xray: endotracheal tube in appropriate position  Endoscopy in ICU: bleeding esophageal varices status post banding and bleeding duodenal ulcer status post endoscopic intervention | Expected Learner Actions:  Call critical care to transfer the patient to the ICU.  GI performs bedside endoscopy. | Modifiers  If team does not call critical care, the nurse will prompt team regarding disposition.  Triggers  For progression to the next step: transfer the patient to the ICU**,** GI performs endoscopy. |
| **3 days: Stable on medical floor**  BP 108/74, HR 96, RR 16, SpO2 98% on RA, T 98 | **Patient:** comfortable, no acute distress  Hemoglobin 10.8 | Expected Learner Actions:  Repeat CBC to monitor hemoglobin.  Order nonselective beta blocker (carvedilol, nadolol, propranolol).  Order oral PPI twice daily. | Modifiers  If the team doesn’t order appropriate outpatient medications, the GI fellow will call to ensure the patient is discharged on appropriate medications.  Triggers  Ordering appropriate outpatient medications will trigger the end of the case. |

**Ideal Scenario Flow**

The learners enter the room to find the patient hypotensive, tachycardic, and actively vomiting blood with an estimated blood loss of 500cc. Based on the history of chronic ibuprofen use and alcohol use disorder with new onset ascites, their differential should include a brisk upper GI bleed secondary to esophageal varices rupture and/or peptic ulcer disease. The team obtains appropriate access (2 large bore IVs or central access), orders labs (CBC, BMP, LFTs, coagulation studies, fibrinogen, venous blood gas with lactate, type and screen) and orders IV fluids and blood transfusion products. The learners will start IV octreotide, IV Protonix (bolus followed by continuous or intermittent dosing) and ceftriaxone. GI will then be consulted for endoscopy for brisk upper GI bleed likely secondary to a ruptured varix or ulcer. The patient is then intubated for airway protection and transferred to the ICU for further care. GI performs a bedside endoscopy in the ICU and finds an acute variceal bleed and duodenal ulcer, both of which require endoscopic intervention. The patient is stabilized, transferred to the general medical floor, and discharged on a nonselective beta blocker and oral PPI twice daily with GI follow up.

**Anticipated Management Mistakes**

1. *Failure to recognize a variceal bleed*: Learners had to read the patient’s chart and ask the patient to disclose his alcohol use, which caused some learner groups to not put variceal bleed on their differential diagnosis. This can be minimized with orientation to the simulation lab and available materials for use.
2. *Not starting octreotide*: Octreotide decreases portal venous pressures and therefore decreases the risk of variceal bleeding. It should be started in this patient with a past medical history of alcohol use disorder and new onset ascites who developed acute upper GI bleeding. This information was included in the debriefing PowerPoint.
3. *Not starting high dose IV PPI*: High dose IV PPIs (Protonix 80mg IV BID or 80mg IV bolus followed by a drip) should be used in cases of suspected upper GI bleed. Indications for PPI use was outlined in the debrief session.
4. *Not starting ceftriaxone*: Patients with ascites and GI bleed are at risk of developing SBP and should be started on prophylaxis, which can include a regimen of ceftriaxone 1g IV daily. This information was included in the debriefing PowerPoint.
5. *Failure to recognize hemorrhagic shock:* Early stages of hemorrhagic shock include tachycardia, even if mild. We found that hypotension was needed for learners to recognize shock. Debriefing materials included the stages of hemorrhagic shock to educate learners on this topic.
