# Supplementary material for: Acute Upper Gastrointestinal Bleeding: A Hands-On Simulation Case for Internal Medicine Residents Improves Knowledge and Confidence
Source: MedEdPORTAL. 2025 Aug 1;21:11541. doi: 10.15766/mep_2374-8265.11541 (PMC12313986; doi:10.15766/mep_2374-8265.11541)
Supplement: Supplementary file 1 — Simulation Case.docxPatient HPI, Labs, and Imaging.pptxPre- and Postsimulation Surveys.docxFaculty Guide.docxDebriefing.pptxCritical Action Checklist.docx [file mep_2374-8265.11541-s001.zip › B. Patient HPI, Labs, and Imaging.pptx]

## Slide 1
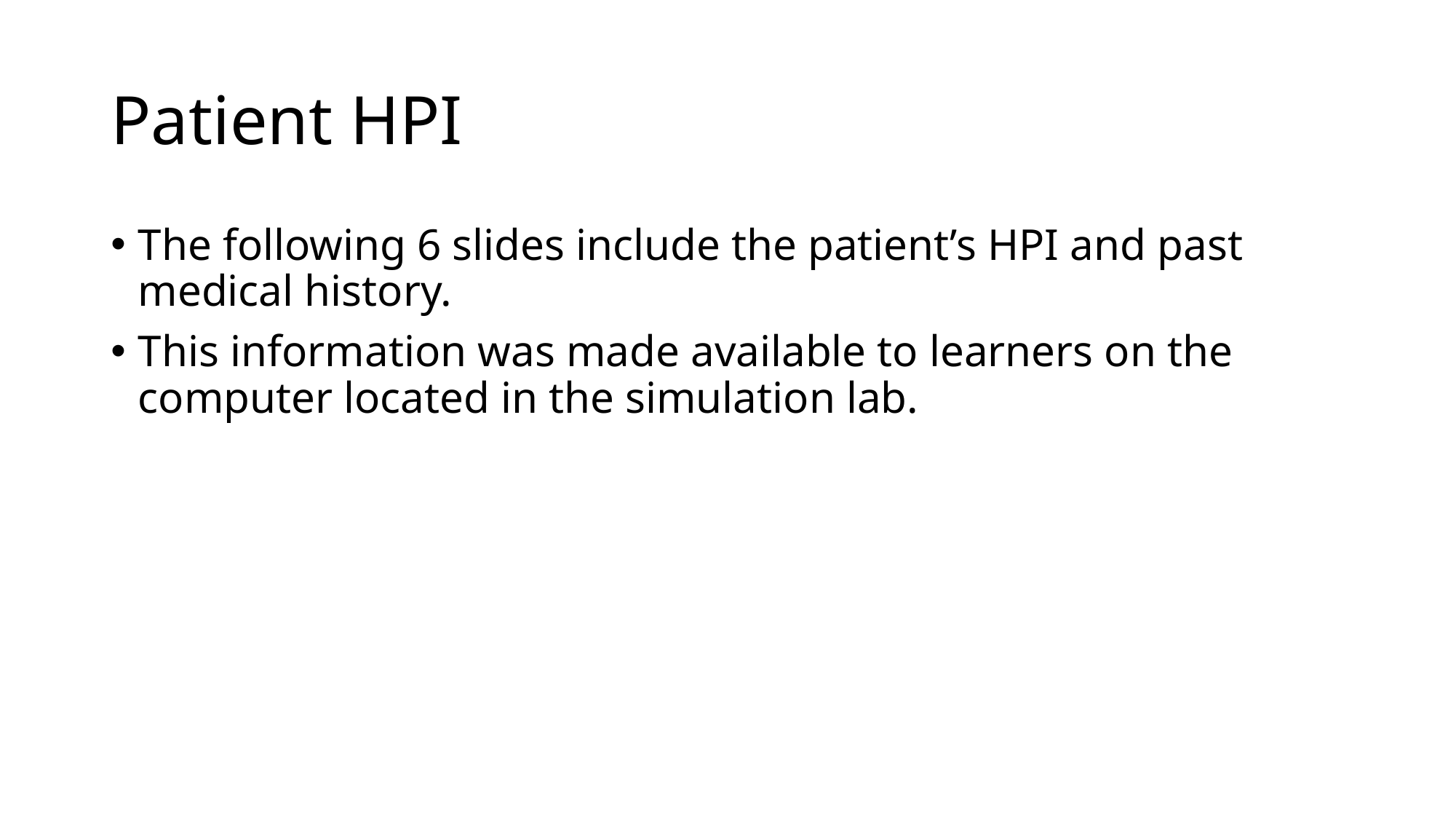

# Patient HPI
The following 6 slides include the patient’s HPI and past medical history.
This information was made available to learners on the computer located in the simulation lab.

## Slide 2
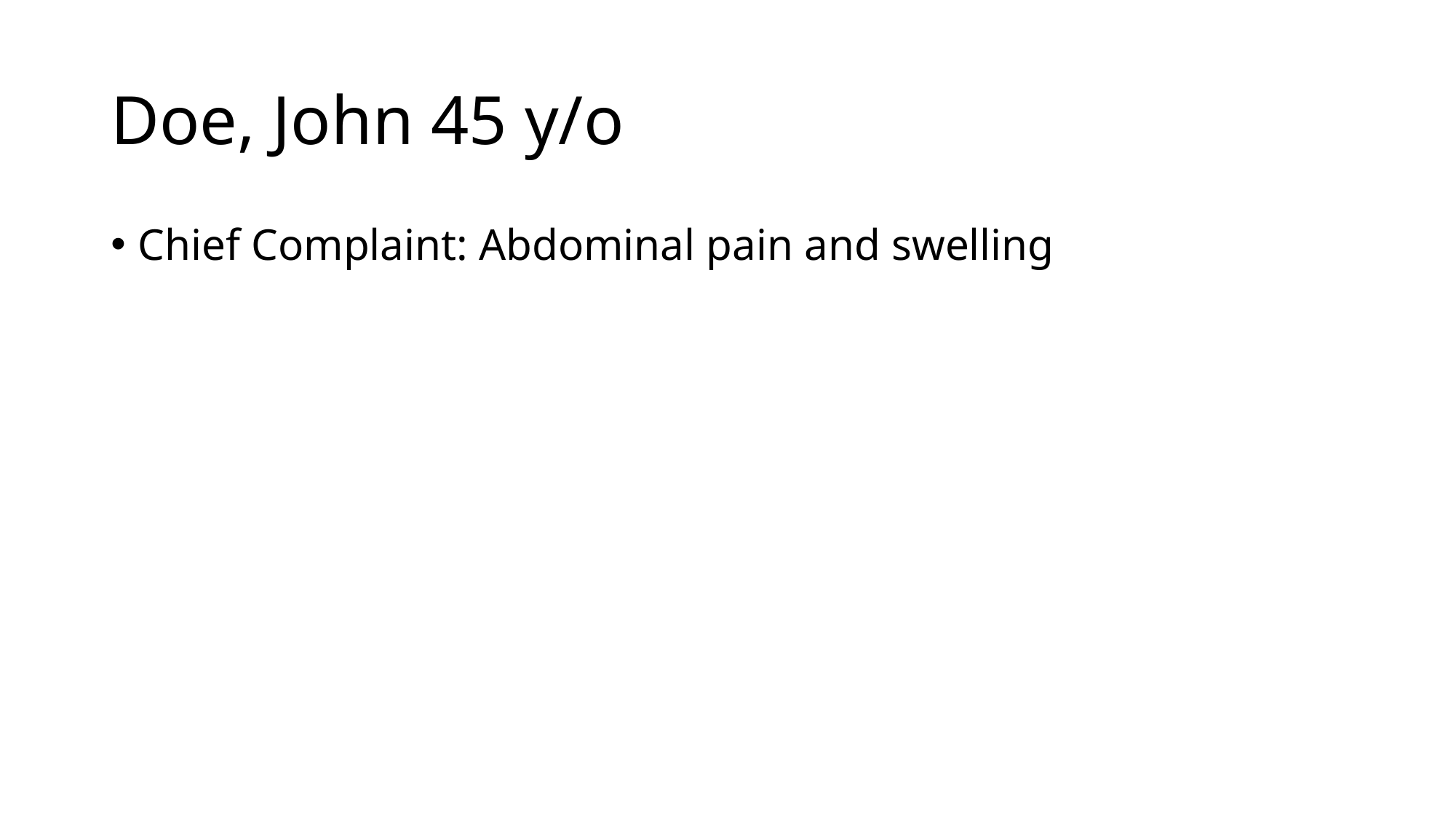

# Doe, John 45 y/o
Chief Complaint: Abdominal pain and swelling

## Slide 3
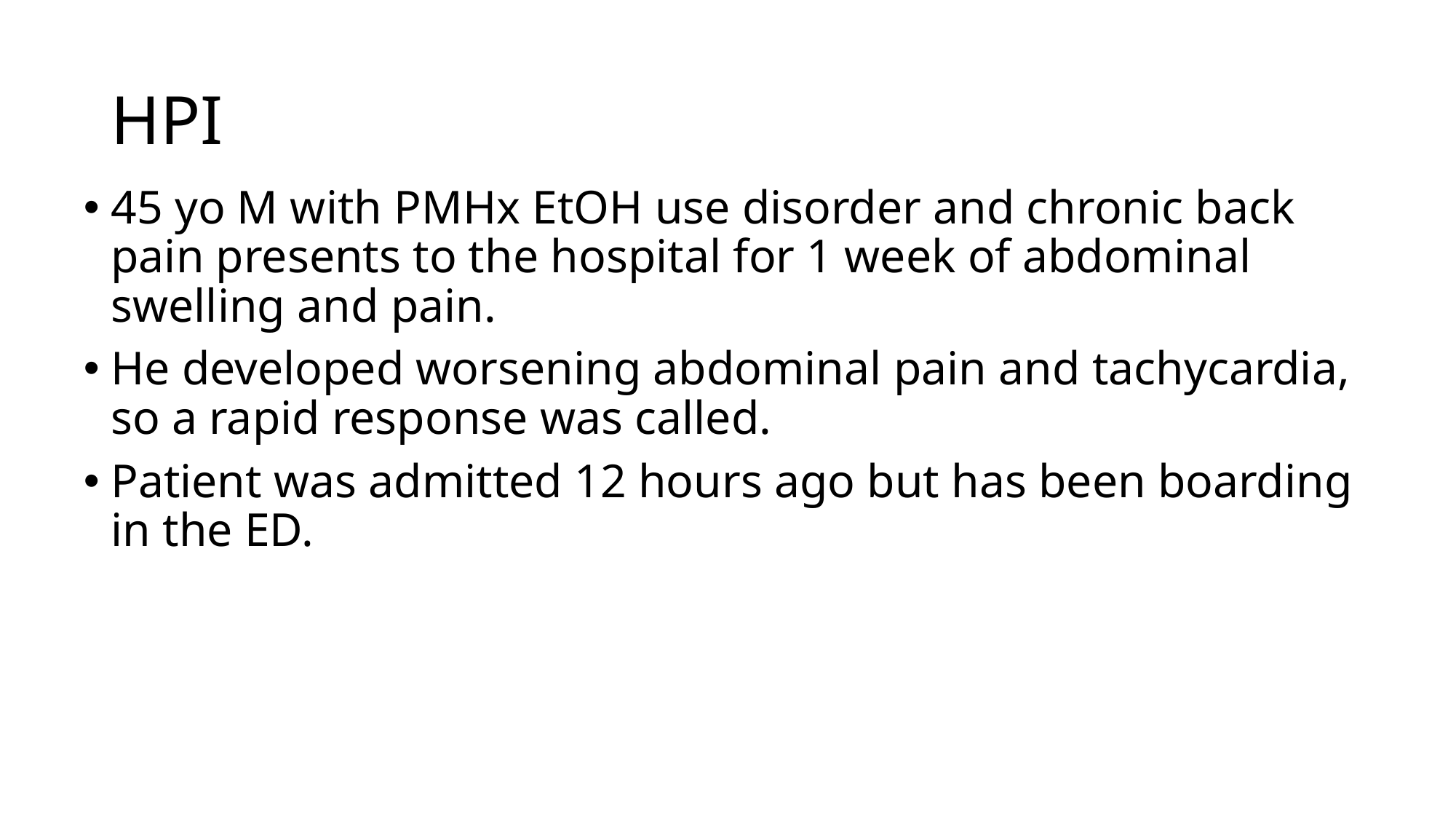

# HPI
45 yo M with PMHx EtOH use disorder and chronic back pain presents to the hospital for 1 week of abdominal swelling and pain.
He developed worsening abdominal pain and tachycardia, so a rapid response was called.
Patient was admitted 12 hours ago but has been boarding in the ED.

## Slide 4
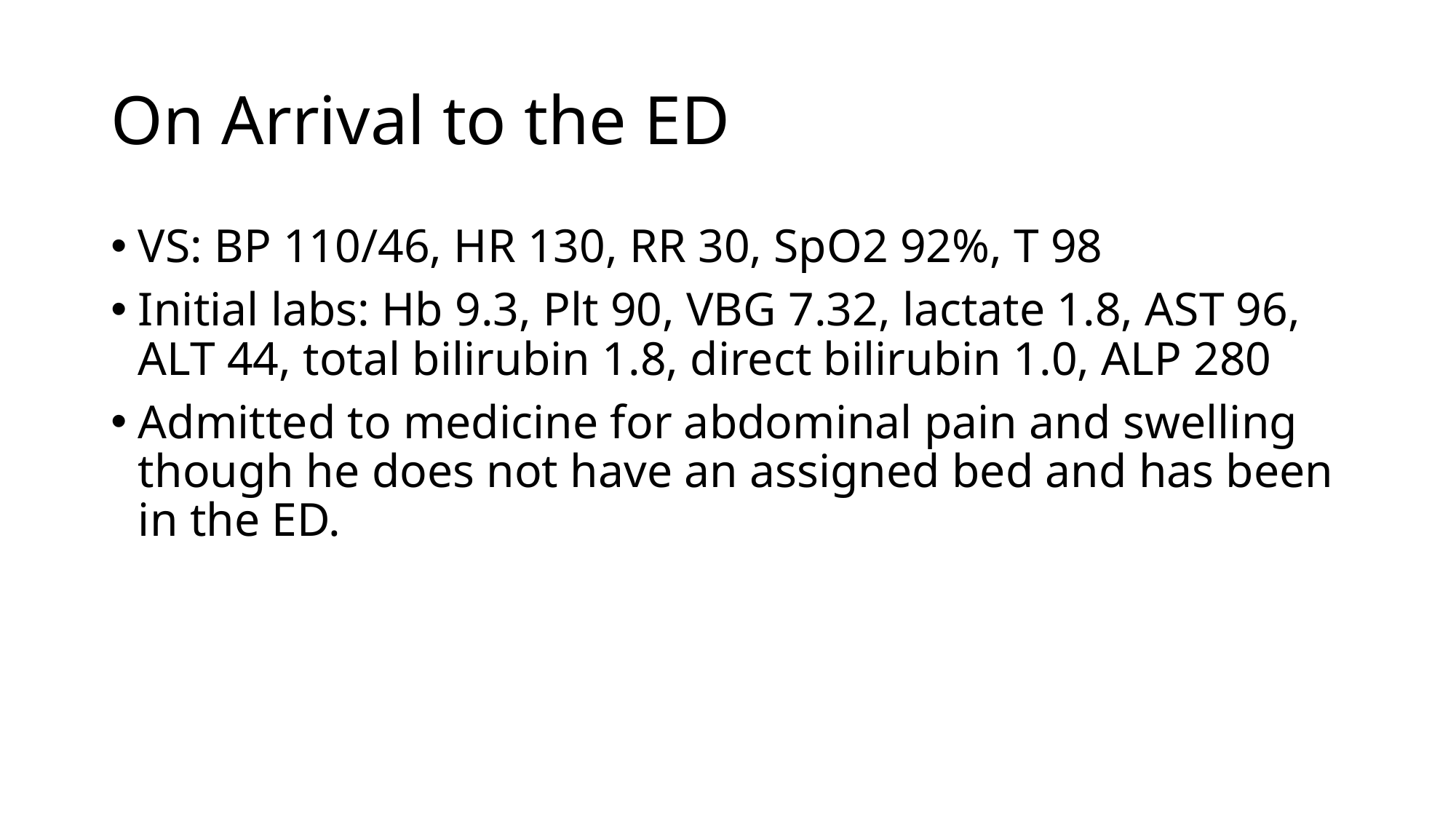

# On Arrival to the ED
VS: BP 110/46, HR 130, RR 30, SpO2 92%, T 98
Initial labs: Hb 9.3, Plt 90, VBG 7.32, lactate 1.8, AST 96, ALT 44, total bilirubin 1.8, direct bilirubin 1.0, ALP 280
Admitted to medicine for abdominal pain and swelling though he does not have an assigned bed and has been in the ED.

## Slide 5
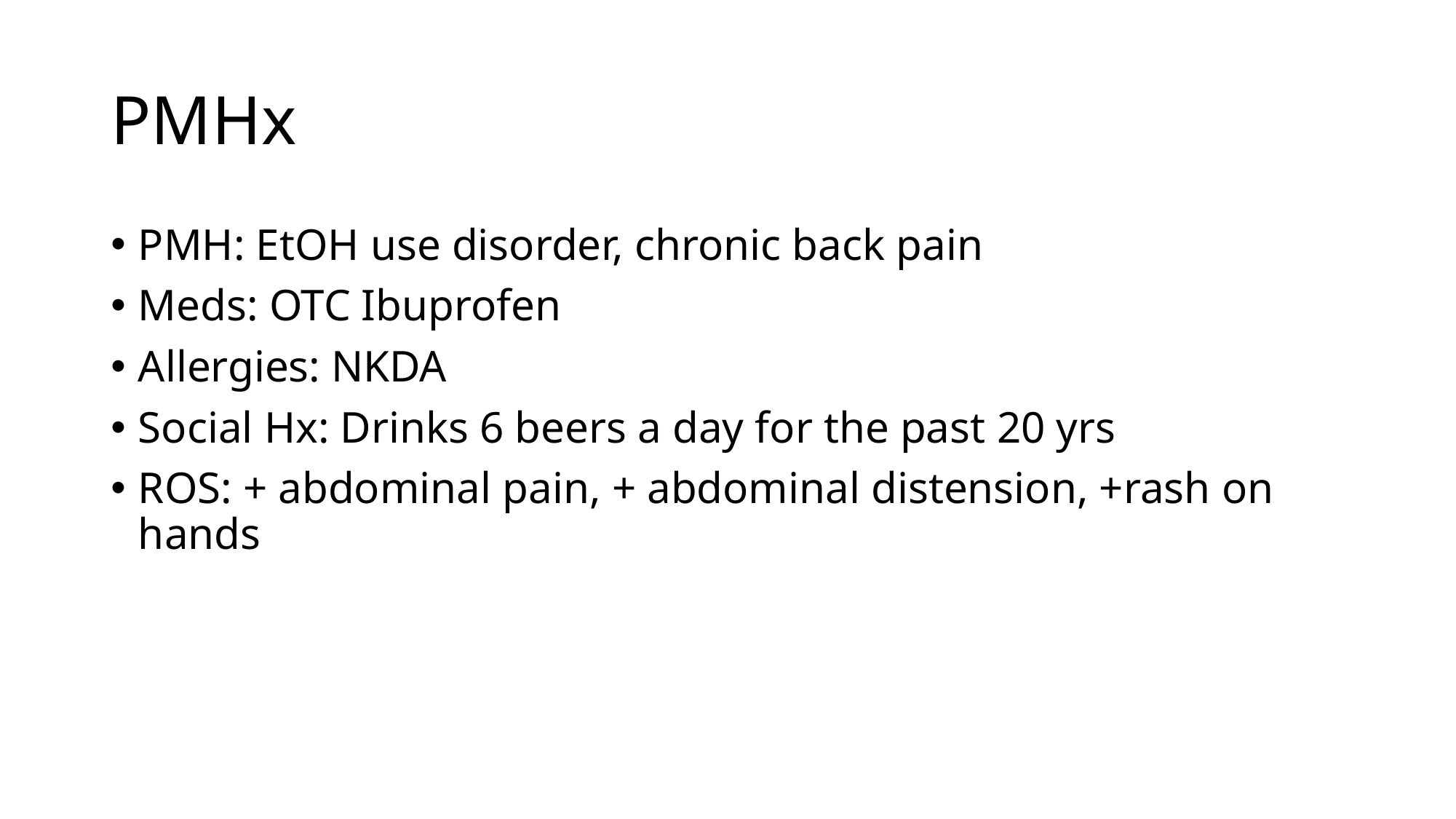

# PMHx
PMH: EtOH use disorder, chronic back pain
Meds: OTC Ibuprofen
Allergies: NKDA
Social Hx: Drinks 6 beers a day for the past 20 yrs
ROS: + abdominal pain, + abdominal distension, +rash on hands

## Slide 6
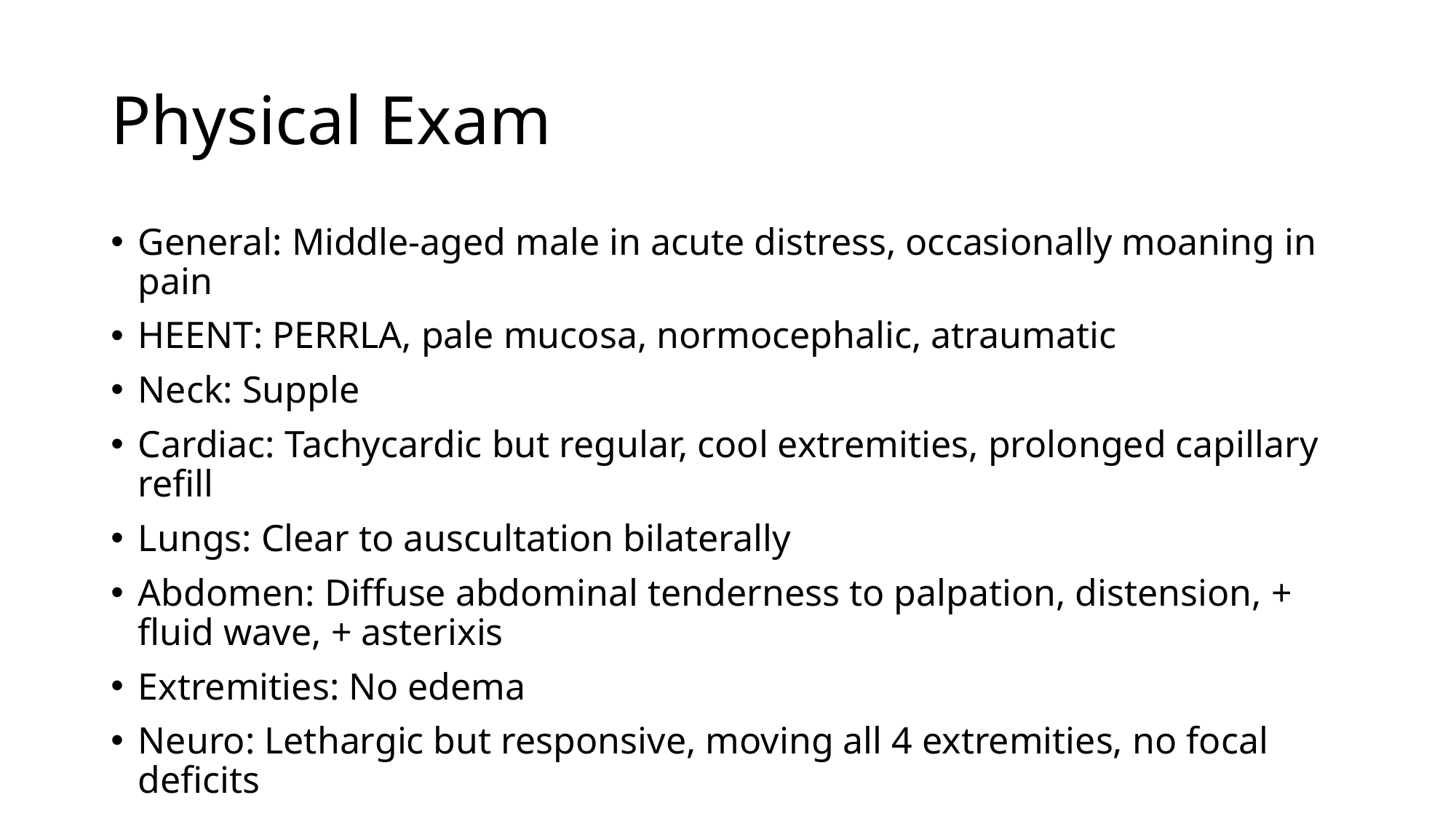

# Physical Exam
General: Middle-aged male in acute distress, occasionally moaning in pain
HEENT: PERRLA, pale mucosa, normocephalic, atraumatic
Neck: Supple
Cardiac: Tachycardic but regular, cool extremities, prolonged capillary refill
Lungs: Clear to auscultation bilaterally
Abdomen: Diffuse abdominal tenderness to palpation, distension, + fluid wave, + asterixis
Extremities: No edema
Neuro: Lethargic but responsive, moving all 4 extremities, no focal deficits
Skin: Pale, + spider angioma, + palmer erythema

## Slide 7
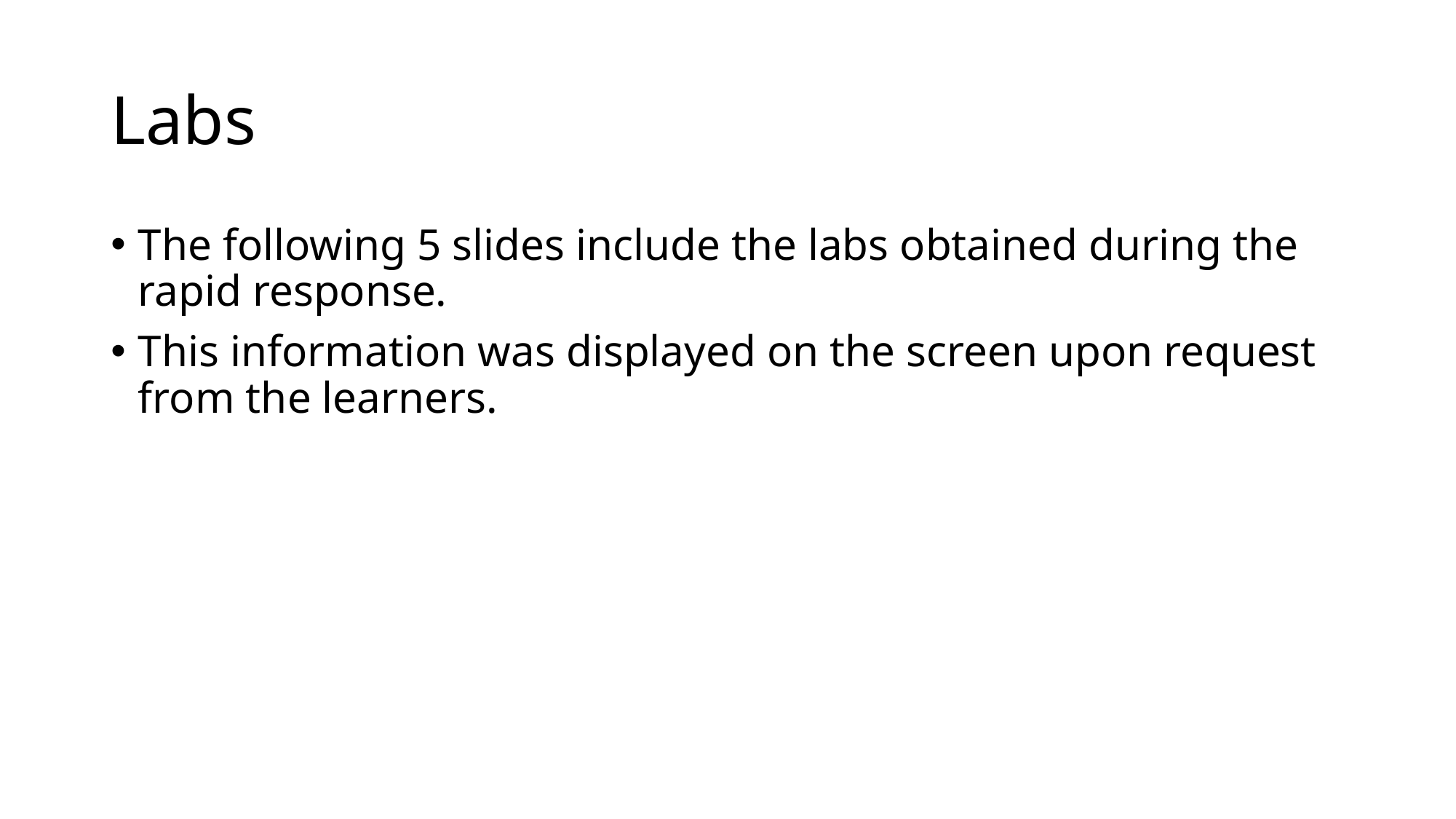

# Labs
The following 5 slides include the labs obtained during the rapid response.
This information was displayed on the screen upon request from the learners.

## Slide 8
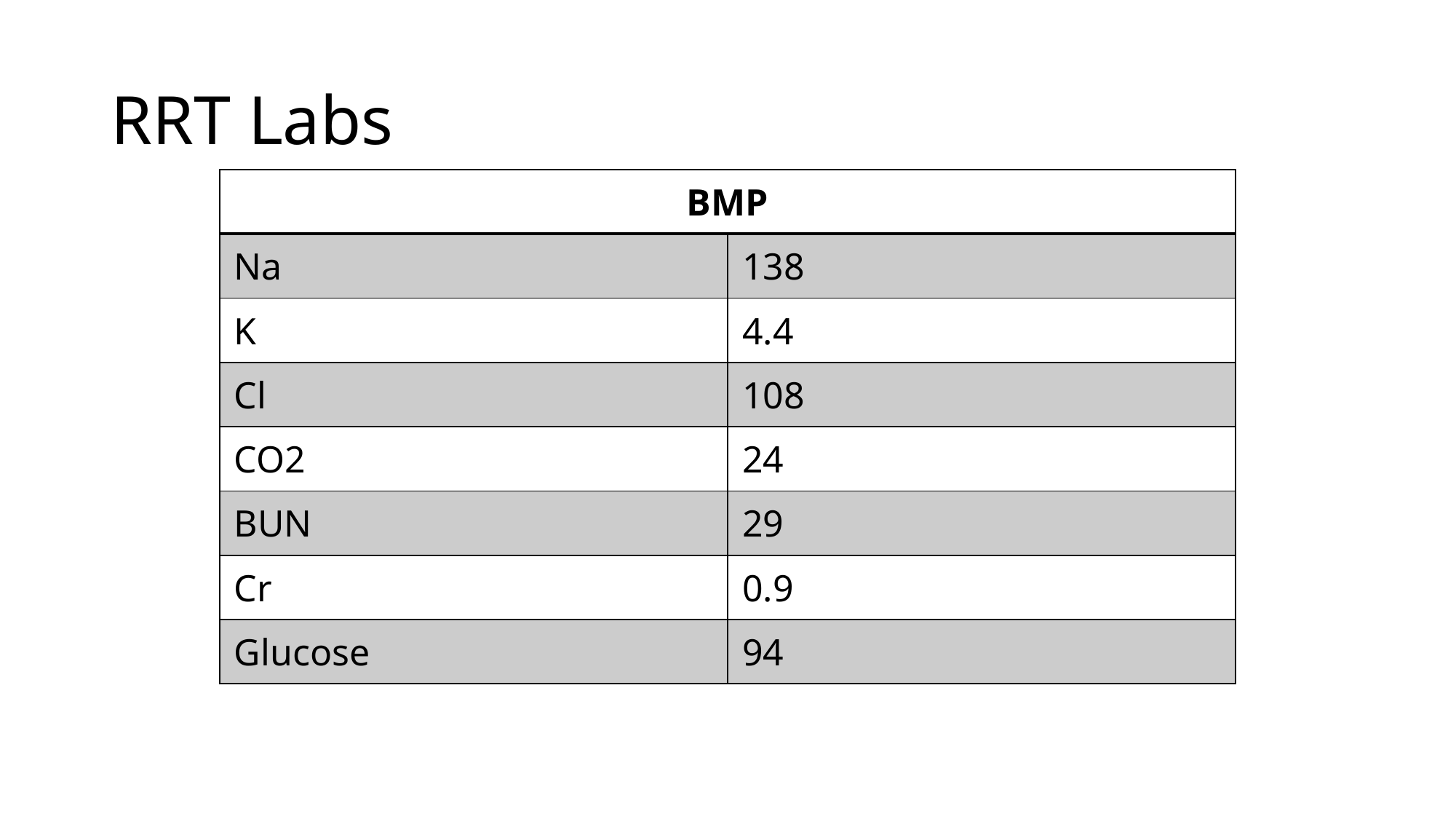

# RRT Labs
| BMP | |
| --- | --- |
| Na | 138 |
| K | 4.4 |
| Cl | 108 |
| CO2 | 24 |
| BUN | 29 |
| Cr | 0.9 |
| Glucose | 94 |

## Slide 9
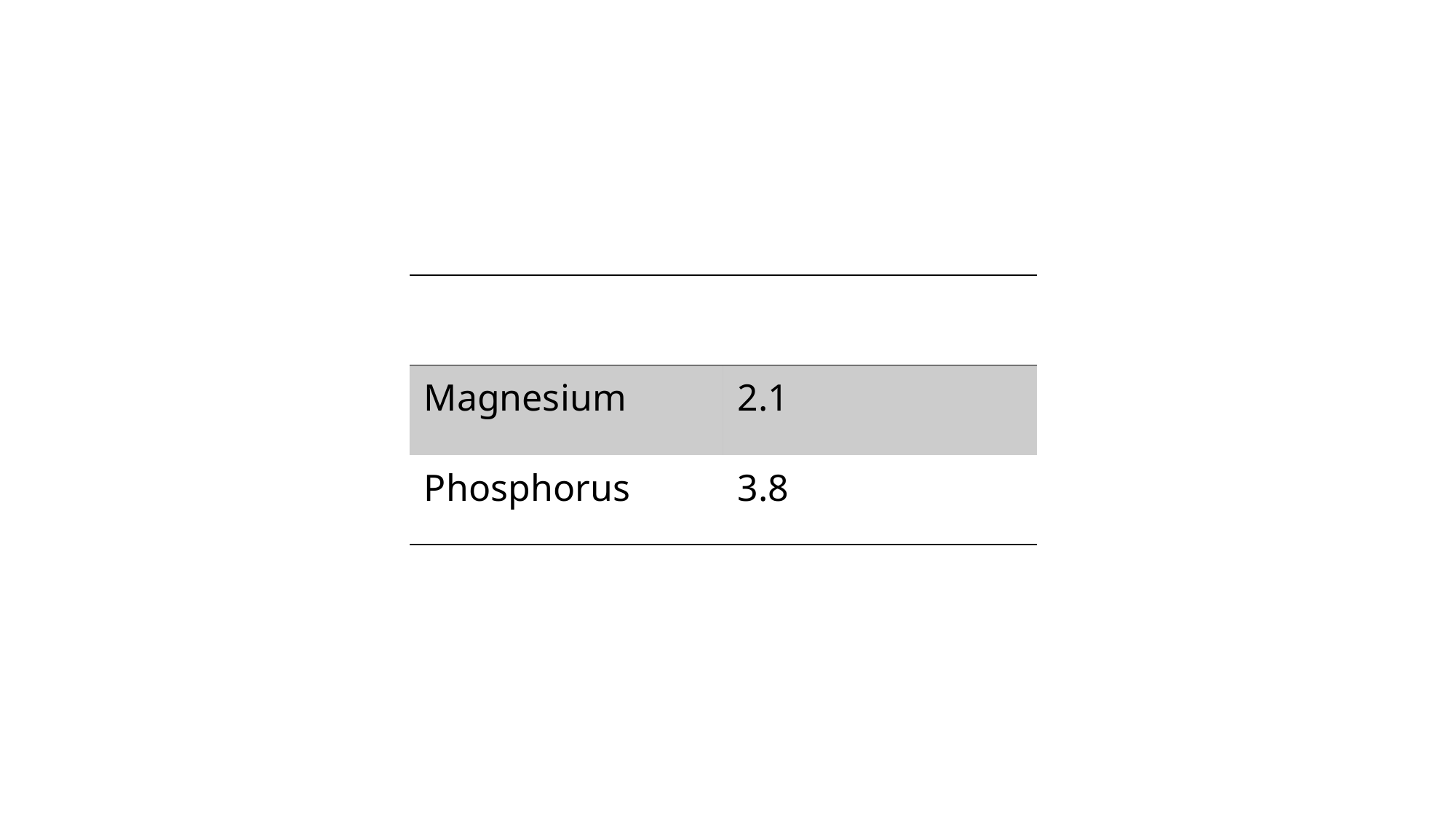

| | |
| --- | --- |
| Magnesium | 2.1 |
| Phosphorus | 3.8 |

## Slide 10
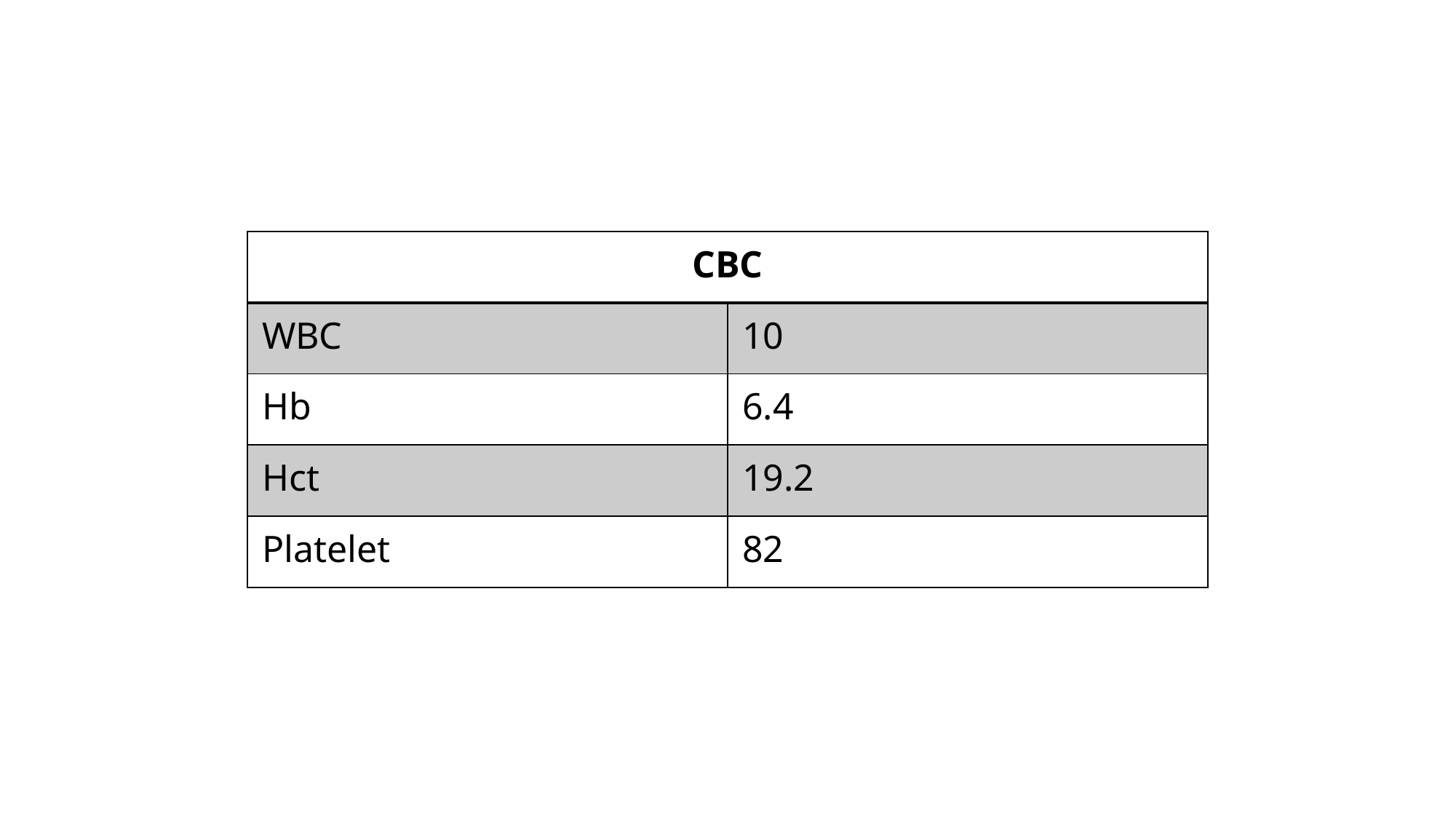

| CBC | |
| --- | --- |
| WBC | 10 |
| Hb | 6.4 |
| Hct | 19.2 |
| Platelet | 82 |

## Slide 11
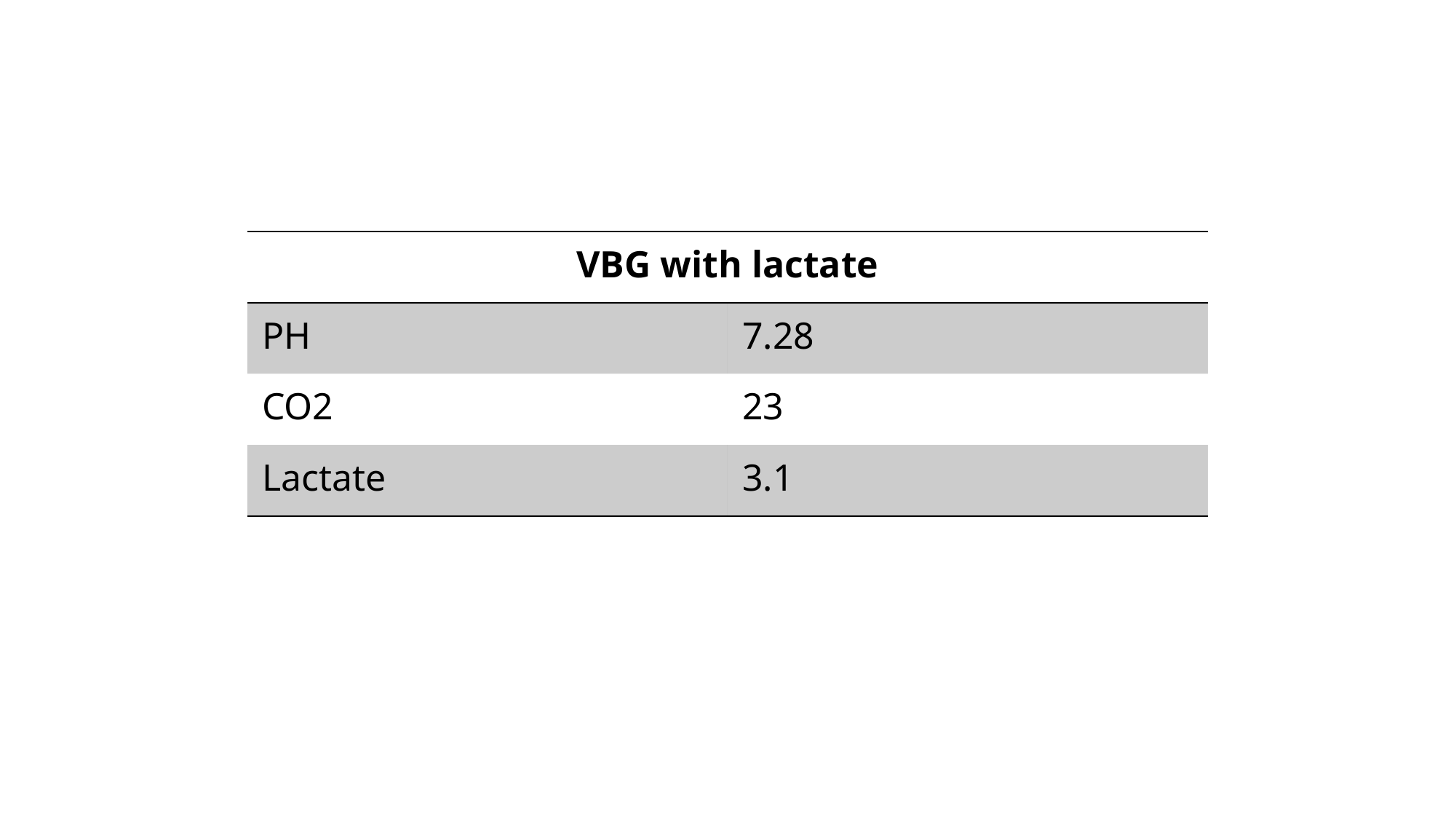

| VBG with lactate | |
| --- | --- |
| PH | 7.28 |
| CO2 | 23 |
| Lactate | 3.1 |

## Slide 12
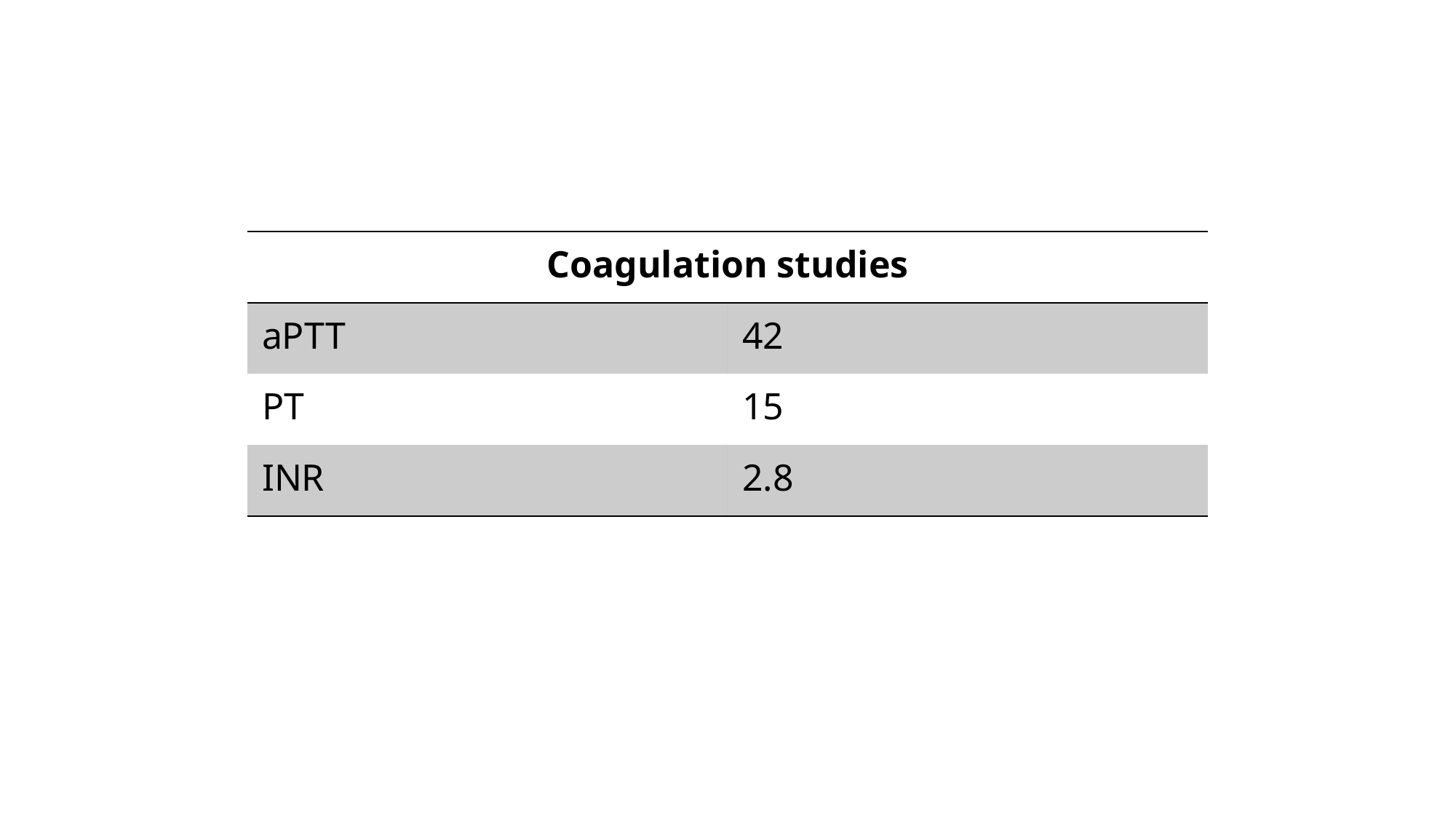

| Coagulation studies | |
| --- | --- |
| aPTT | 42 |
| PT | 15 |
| INR | 2.8 |
